# MAVS as a Key Regulator of Tumor Proliferation, Survival, the Tumor Microenvironment, and Immunity

**DOI:** 10.3390/biom16040501

**Published:** 2026-03-26

**Authors:** Sweta Trishna, Anna Shteinfer-Kuzmine, Vered Chalifa-Caspi, Varda Shoshan-Barmatz

**Affiliations:** 1 Department of Life Sciences, Ben-Gurion University of the Negev, Beer Sheva 84105, Israel; shweta.trishna@gmail.com; 2 National Institute for Biotechnology in the Negev, Ben-Gurion University of the Negev, Beer Sheva 84105, Israel; shteinfe@post.bgu.ac.il; 3 Ilse Katz Institute for Nanoscale Science and Technology, Ben-Gurion University of the Negev, Beer Sheva 84105, Israel; veredcc@bgu.ac.il

**Keywords:** cancer, immunity, mitochondria, MAVS

## Abstract

The mitochondrial anti-viral signaling protein, MAVS, is a central regulator of innate anti-viral immunity. Recently, we demonstrated that MAVS is overexpressed in cancer, where its downregulation resulted in reduced cell proliferation and the expression and nuclear translocation of proteins associated with transcriptional regulation and inflammation. In this study, we demonstrate that CRISPR/Cas9-mediated MAVS depletion in PC-3 prostate cancer cells suppresses proliferation, disrupts immune evasion, and alters the tumor microenvironment. Proteomic profiling of the MAVS-KO cells by LC-MS/MS revealed changes in the expression of proteins associated with immunity, cell signaling, mitochondrial function, metabolism, protein synthesis and degradation, and epigenetic regulation. In contrast to MAVS-expressing cells, MAVS-KO cells implanted subcutaneously in mice formed very small tumors. This inhibited tumor growth was linked to reduced proliferation, and enhanced apoptosis, as indicated by strong TUNEL staining and elevated activated caspase-3. Importantly, the small “tumors” derived from MAVS-KO cells displayed a distinct morphology: diminished cancer stem-cell populations, an altered tumor microenvironment and inflammatory response, increased immune cell infiltration, and reduced PD-L1 expression. Together, these findings establish MAVS as a key mediator of cancer-cell survival, inflammation, and immune regulation, and, thus, its upregulation in tumors makes it a potential anti-cancer target.

## 1. Introduction

Mitochondria, the powerhouse of the cell, are the signaling hub for regulating metabolism apoptosis, the cell cycle, proliferation, differentiation, epigenetics, immune signaling, and aging processes [1]. Mitochondria also serve as integrators and amplifiers of programed cell death [2].

Mitochondrial signaling is necessary for responses to activators of innate immune signaling, and mitochondrial signaling dictates macrophage polarization and function [3,4]. The importance of the mitochondria to cellular and organismal homeostasis is reflected by the association of mitochondrial dysfunction with a wide range of human pathologies, including neurodegenerative and metabolic diseases, aging, and cancer [5].

One of the links between the mitochondria and immune signaling is the mitochondrial anti-viral-signaling (MAVS) protein, also known as IFN-β promoter stimulator I (IPS-1), caspase activation recruitment domain adaptor inducing I FN-β (CARDIF), or the virus-induced signaling adaptor (VISA) [6,7]. MAVS, at the outer mitochondrial membrane (OMM), plays a central role in initiating the anti-viral innate immune response in mitochondria [8,9,10,11], triggered by viral RNA recognition through retinoic acid-inducible gene I (RIG-I) and other RIG-I-like receptors (RLRs) [12].

MAVS is a 540 amino acid protein composed of three main domains: an N-terminal caspase activation and recruitment domain (CARD), a proline-rich region, and a C-terminal transmembrane domain [7]. The transmembrane domain anchors MAVS to the outer mitochondrial membrane and is essential for its antiviral function [9,10]. The N-terminal CARD allows MAVS to interact with the CARDs of RLRs, such as RIG-I and MDA5 [8,9]. Upon activation, MAVS forms a large oligomeric complex that serves as a signaling platform [6,13] to recruit downstream molecules and kinases, ultimately leading to the production of type I interferon (IFN) proteins and pro-inflammatory cytokines [8,9,10].

MAVS activation leads to elevated levels of pro-inflammatory cytokines through the stimulation of key transcription factors, including nuclear factor κB (NF-κB), interferon regulatory factor 1 (IRF1), and interferon regulatory factor 3 (IRF3) [14]. MAVS also interacts with mitofusin 2 (Mfn2), which prevents it from binding to cytosolic proteins such as RIG-I and MDA5 [14]. Although the complete mechanisms governing MAVS’ signal transduction and regulation are not fully understood, its activation on the mitochondria, endoplasmic reticulum (ER), and peroxisomes are essential for mounting a robust anti-viral innate immune response [15]. MAVS has been proposed to bind to the mitochondrial-associated membrane (MAM) [16], allowing for inter-organelle communication that regulates stress and metabolic signaling.

MAVS protein is ubiquitously expressed, with higher levels in the heart, skeletal muscles, liver, placenta, and peripheral blood leukocytes [17]. It is also expressed in various viral non-infected cancer cell lines and patient-derived tumors, including lung, liver, bladder, and cervical cancers, and its role has been studied in cancer [18].

Recent studies have revealed that, beyond its well-established role in antiviral defense, MAVS has emerged as a key regulator of diverse physiological and metabolic processes. MAVS is involved in host responses to bacterial and parasitic infections, and has been linked to a wide range of pathological conditions, including cancer, systemic lupus erythematosus, and kidney and cardiovascular diseases [17,19]. These findings highlight the critical role of MAVS in maintaining immune homeostasis and underscore its relevance across multiple disease contexts, thereby opening new avenues for therapeutic intervention [20,21].

The mechanisms linking MAVS to various diseases are complex and involve dysregulated calcium homeostasis, metabolic reprogramming, iron imbalance, oxidative stress, inflammation, impaired mitochondrial dynamics, and impaired mitochondrial autophagy. In all the above factors and associated diseases, the mitochondria and their gatekeepers, the voltage-dependent anion channel 1 (VDAC1), are involved [22,23,24].

Recently, we demonstrated that MAVS directly interacts with VDAC1, decreasing its conductance, and identified the VDAC1 binding site in MAVS [18]. MAVS has been shown to interact with VDAC1 [18] and to modulate VDAC1 protein stability through the ubiquitin–proteasome pathway and to induce apoptosis by caspase-3 activation [25].

VDAC1 stands at the crossroads between mitochondrial energy production and metabolism, Ca^2+^ homeostasis, apoptosis execution and regulation, and other stress-associated processes [22,23,26,27]. VDAC1 overexpression, its oligomerization, and apoptosis induction have been associated with different diseases, including neurodegenerative disease, type 2 diabetes, lupus, colitis, acute liver injury, rheumatoid arthritis, spinal cord injury, and myocardia in humans and rats [27,28]. Mice deficient in MAVS are impaired in their ability to produce type I IFNs, making them highly susceptible to RNA virus infections [29]. Research has revealed that both partial and complete MAVS deficiency in mice leads to decreased cardiac function and enlarged hearts in mice due to disruptions in mitochondrial function, energy production, and lipid metabolism [30].

In our previous study [18], using MAVS-specific siRNA, we explored MAVS’ role in promoting chronic inflammation and proliferation in cancer and contributing to immune evasion. Silencing MAVS expression reduced cell proliferation and the expression and nuclear translocation of proteins associated with transcriptional regulation. This also reduced inflammasome components, inhibiting their activation/assembly.

In this study, we evaluate the impact of MAVS downregulation in a peritumoral context using CRISPR/Cas9-mediated knockout (MAVS-KO) in PC-3 prostate cancer cells. MAVS depletion inhibited cell proliferation, and, in mice, MAVS-deficient cells formed only very small tumors with inhibited proliferation and an altered microenvironment and immunity. Proteomic analysis revealed changes in proteins regulating immunity, signaling, mitochondrial function, metabolism, and epigenetics, highlighting the multifunctional role of MAVS in tumor cells.

## 2. Materials and Methods

### 2.1. Materials

Paraformaldehyde, hematoxylin and eosin, Trypan blue, Triton X-100, and Tween-20, 4′,6-diamidino-2-phenylindole (DAPI), Crystal violet and Tris were obtained from Sigma Aldrich (St. Louis, MO, USA). Roswell Park Memorial Institute (RPMI), normal goat serum (NGS), and the supplements fetal bovine serum (FBS), L-glutamine, and penicillin-streptomycin and phosphate-buffered saline (PBS) were obtained from Gibco (Grand Island, NY, USA). A chemiluminescence detection kit for HRP were obtained from Advantsa (San Jose, CA, USA). A protease inhibitor cocktail set III, EDTA-free was purchased from Millipore (Billerica, MA, USA). JetPrime transfection reagent was obtained from PolyPlus transfection (Illkirch, France). The primary and HRP-conjugated and fluorophore-conjugated secondary antibodies, their sources, and the dilutions used are detailed in Supplementary Table S1.

### 2.2. Cell Culture and CRISPR/Cas9 MAVS Knockout

PC-3 (prostate adenocarcinoma) cells were from the American Type Culture Collection (ATCC) (Manassas, VA, USA). Cells were maintained in ATCC-recommended medium at 37 °C in an incubator with 5% CO_2_. Cells were routinely tested for mycoplasma contamination.

MAVS CRISPR/Cas9 Knockout Plasmid with GFP marker (sc-432790) was purchased from Santa Cruz Biotechnology (Dallas, TX, USA). PC-3 cells were seeded in 6-well cell-culture plates (200,000 cells/well) and allowed to attach overnight. Cells were transfected with the knockout plasmid, as per the manufacturer’s instructions, using JetPrime transfection reagent (PolyPlus transfection; Illkirch, France). GFP-positive cells were sorted using FACS (SY3200 cell sorter, Sony Biotechnology, San Jose, CA, USA) and plated in 96-well plates (1 cell/well). Cells were grown for ten days, and each colony was transferred to a separate well of 12-well cell-culture plates. Individual colonies with MAVS-KO were selected for maintenance after immunoblotting for MAVS.

### 2.3. Protein Extraction, Gel Electrophoresis and Immunoblotting

Cells were harvested and washed twice with ice-cold PBS, and the pellets were lysed on ice for 30 min in a lysis buffer (50 mM Tris-HCl, pH 7.5, 150 mM NaCl, 1 mM EDTA, 1.5 mM MgCl_2_, 10% glycerol, 1% Triton X-100), freshly supplemented with a protease inhibitor cocktail Millipore (Billerica, MA, USA), incubated on ice for 20 min, and centrifuged (10 min, 12,000× *g*).

Tumors were lysed in a lysis buffer (4% SDS, 100 mM Tris-HCl, pH 8.0, 5 mM DTT) supplemented with 0.5 mM EGTA and 0.5 mM PMSF. Then tumor tissues were homogenized and incubated for 3 min at 95 °C, sonicated, and centrifuged (10 min, 15,000× *g*, room temperature (RT)).

The protein concentration of the supernatant was determined, and cells were stored at −80 °C until used for gel electrophoresis and immunoblotting. Protein samples (10–20 μg) were subjected to SDS-PAGE and were then electro-transferred onto nitrocellulose membranes for immunostaining. The membranes were incubated with a blocking solution containing 5% non-fat dry milk and 0.1% Tween-20 in tris-buffered saline (TBST), followed by incubation with primary antibodies (Table S1). Subsequently, membranes were incubated with HRP-conjugated anti-mouse or anti-rabbit IgG as secondary antibodies (Table S1). Band intensities were visualized using FUSION-FX (Vilber Lourmat; Marne-la-Vallée, France) and quantified using ImageJ (version 1.54p; NIH, Bethesda, MD, USA) software, and the values were normalized to the intensities of the appropriate signal of a loading control.

### 2.4. In Vitro Clonogenic Assay

A cell colony assay was performed with crystal violet. PC-3 cells both expressing and MAVS-KO were seeded in 6-well plates (0.5 × 10^5^ cells/well) and cultured with normal growth medium for 10 or 14 days. Then, the medium was decanted and the colonies were fixed with 4% paraformaldehyde at RT for 10 min, and stained with 0.5% crystal violet (in 20% methanol) for 30 min for colony visualization. Plates were then washed with water, dried, and imaged with a wide-field microscope (Olympus, Tokyo, Japan). Then, crystal violet was extracted from cells using methanol (100%), and the color intensity was determined by measuring absorbance at 595 nm using a spectrophotometer.

### 2.5. Immunofluorescence (IF) of Cells

IF staining of cells was performed in cells plated on sterile glass coverslips placed in 12-well cell culture plates (3 × 10^4^ cells/well), incubated overnight in CO_2_ incubator washed with PBS, and fixed with 4% paraformaldehyde. To reduce non-specific binding, cells were incubated with 5% normal goat serum for 2 h, and then incubated with primary antibodies (Table S1) overnight at 4 °C. The next day, the PBST-washed samples were incubated with fluorescent-tagged secondary antibodies (Table S1) for 2 h at RT in the dark. Following a wash with PBS, the samples were incubated with DAPI for 15 min in the dark, washed, mounted with Fluoroshield mounting medium (ImmunoBioScience, Mukilteo, WA, USA), and viewed by confocal microscopy (Olympus 1X81; Olympus corporation; Tokyo, Japan).

### 2.6. Xenograft Mouse Models

Ten athymic 6-week-old male SCID nude mice (weight ~20–25 g) were obtained from Envigo (Indianapolis, IN, USA) and allowed a week of acclimatization to their new surroundings. Control or MAVS-KO PC-3 cells (2 × 10^6^) were injected subcutaneous (s.c.) into the hind leg flanks of the mice (5 mice per group). After 15 days after inoculation, the developing tumors were measured in a blind manner in two dimensions with a digital caliper, and tumor volume was calculated as follows: volume = (X^2^ × Y)/2, where X and Y are the short and long tumor dimensions, respectively.

Beginning on the day of inoculation, mouse weight and tumor volume were monitored twice a week for a period of 41 days using a digital caliper. At the end point of the experiment, i.e., when tumor volumes reached ~2100 mm^3^, the mice were sacrificed using CO_2_ gas. The tumors were excised and ex vivo weight was determined, and half of each tumor was either fixed and processed for immunohistochemistry (IHC), immunofluorescence (IF), or frozen in liquid nitrogen for later immunoblot.

All experiments were conducted in accordance with ethical committee guidelines and were approved by the Ben-Gurion University and the State of Israel Institutional Animal Care and Use Committees (approval Code: IL-57-09-2023C; September 2023). In compliance with animal welfare regulations, the maximum allowable tumor burden was limited to approximately 10% of the mouse’s total body weight.

### 2.7. Immunofluorescence (IF) of Tissues

Formalin-fixed and paraffin-embedded 5 μm thick tumor tissue sections were deparaffinized by heating the slides on a 60 °C hot plate for 1 h and using xylene. Thereafter, the sections were rehydrated using a graded ethanol series (100–50%) and subjected to antigen retrieval using either 0.01 M citrate buffer (pH 6.0) or 0.01 M Tris-EDTA (pH 9) at 95–98 °C for 30 min. The sections were incubated in blocking buffer (10% NGS, 1% BSA, and 0.1% Triton) for 2 h and then incubated with primary antibodies in an antibody buffer (5% NGS, 1% BSA) (Table S1) overnight at 4 °C. After washing with PBS containing 0.1% Triton (PBST), sections were incubated with fluorescent-tagged secondary antibodies (Table S1) for 2 h at RT in the dark. Following a wash with PBST, sections were incubated with DAPI for 15 min in the dark, washed, mounted with Fluoroshield mounting medium (Immunobioscience, Mukilteo, WA, USA), and imaged by confocal microscopy (Olympus 1X81).

### 2.8. TUNEL Assay

Fixed tumor sections in paraffin were processed for the terminal deoxynucleotidyl transferase dUTP nick end labeling (TUNEL) assay using the DeadEnd Fluorometric TUNEL system (Promega, Madison, WI, USA) according to the manufacturer’s instructions. Sections were deparaffinized, equilibrated in PBS, permeabilized with proteinase K (20 μg/mL in PBS), post-fixed in 4% paraformaldehyde, and incubated in TdT reaction mix for 1 h at 37 °C in the dark. Slides were then washed in 2× saline-sodium citrate buffer, counter-stained with propidium iodide (PI, 1 µg/mL), and cover slipped with Vectashield mounting medium (Vector Laboratories, Burlingame, CA, USA). Fluorescent images of apoptotic cells (green) and cell nuclei (red) were captured using a confocal microscope (Olympus 1X81, Olympus corporation; Tokyo, Japan).

### 2.9. Sirius Red and Hematoxylin and Eosin Staining

Sirius red staining was performed on fixed and paraffin-embedded tumor sections. The sections were first deparaffinized and rehydrated with a graded ethanol series. Hematoxylin was used for staining the nuclei for 8 min, followed by washing with running tap water. The sections were then incubated with a 0.1% Sirius red-1.3% picric acid solution for one hour. After that, the sections were rapidly washed with 0.5% acetic acid, dehydrated in three changes of 100% ethanol, and cleared in xylene. Finally, the sections were mounted with EUKITT mounting medium (Orsatech, London, UK). The stained sections were photographed with a panoramic scanner (3DHISTECH Ltd., Budapest, Hungary) and quantified using HistoQuant software (Quant Center 2.0 software).

Hematoxylin-eosin (H&E) staining was performed as described previously [31].

### 2.10. Liquid Chromatography–High-Resolution Mass Spectrometry (LC-HR-MS/MS) and Proteomics Analysis

For LC-HR MS/MS analysis, proteins were extracted from CRISPR/Cas9 MAVS-KO and control cells in biological duplicates (*n* = 2 per condition) using lysis buffer (100 mM Tris-HCl, pH 8.0, 5 mM DTT, 4% SDS, and a protease inhibitor cocktail), followed by homogenization, incubation for 3 min at 95 °C, and centrifugation (10 min, 15,000× *g*). The protein concentration of each lysate was determined using a Lowry assay. Samples were stored at −80 °C until LC-HR-MS/MS analysis.

Samples were subjected to tryptic digestion, alkylation, detergent removal, and desalting, and then to LC-HR MS/MS analysis. Mass spectrometry (MS)-based proteomics profiling and initial processing of the results were carried out at the de Botton Institute for Protein Profiling, G-INCPM, at the Weizmann Institute of Science (Rehovot, Israel).

### 2.11. Statistical Analyses for Identification of Differentially Expressed Proteins

LC-HR MS/MS raw data were processed with MaxQuant v2.0.1.0 with the Andromeda search engine. The higher-energy collisional dissociation (HCD) MS/MS spectra were searched against an in silico tryptic digest of Homo sapiens proteins from the UniProt/Swiss-Prot sequence database, including common contaminant proteins. All MS/MS spectra were searched with the following MaxQuant parameters: acetylation (protein N-terminus) and methionine oxidation; cysteine carbamidomethylation as a fixed modification; a maximum two missed cleavages; and precursor mass tolerance set to 4.5 ppm with 20 ppm for fragment spectra. Peptide spectrum matches and proteins were automatically filtered to a 1% false discovery rate based on Andromeda score, peptide length, and individual peptide mass errors. 

Proteins were identified and quantified using the label-free quantification (LFQ method(, yielding a total of 5137 identified protein groups. The initial MS/MS spectra processing and MaxQuant analysis were carried out at the de Botton Institute for Protein Profiling, G-INCPM. Subsequent downstream bioinformatic analysis was performed at the Bioinformatics Core Facility at Ben-Gurion University using R version 4.5.2 (https://github.com/veredcc/Neat_Proteomics, accessed on 17 February 2026).

Proteins marked as “contaminant”, “reverse”, and “only identified by site” were filtered out, as well as those showing zero unique peptides. In an additional filtering step, only proteins in which at least one of the groups (MAVS-KO, control) had two non-zero replicates were retained (*n* = 4638). LFQ intensities were transformed into Log2, and zero intensities were replaced by random numbers derived from a normal distribution in the low expression range (width = 0.2, downshift = 1.8). This imputation approach is based on the Perseus software algorithm, but was re-implemented in R. The imputation process was repeated 10 times to avoid heavy reliance on fabricated numbers. Each of the ten imputed datasets was submitted to hypothesis testing for differential protein expressions using Limma version 3.66.0 [32]. The statistical model tested the contrast: MAVS-KO versus control. Fold change was computed in Log2 scale, but then converted to a linear scale for intuitive biological interpretation. A protein was considered differentially expressed (DE) if it had a nominal *p*-value < 0.01 and absolute fold change (in linear scale) > 1.5 in at least eight of the ten input datasets. This conservative 80% supermajority threshold was chosen over a simple majority (e.g., 6/10) to ensure that the differential expression was driven by underlying biological signal rather than stochastic variability introduced by the imputation process. Hierarchical clustering of the DE proteins, after z-scoring of the imputed Log2-transformed LFQ data, was performed using the pheatmap R function (version 1.0.13), employing Pearson’s dissimilarity and complete linkage.

We performed a functional enrichment analysis of the differentially expressed (DE) protein set. However, no GO terms or pathways remained significant after false discovery rate (FDR) correction. This likely reflects the relatively small number of DE proteins and their functional heterogeneity. Therefore, the proteins were grouped into functional categories based on their literature-supported biological roles.

### 2.12. Statistics

Results are presented as the means ± SEM of results obtained from independent experiments. A difference was considered statistically significant when the *p*-value was <0.05 (*), <0.01 (**), <0.001 (***), or < 0.0001 (****), as assessed through an unpaired Student’s two-tailed *t*-test.

## 3. Results

### 3.1. MAVS-KO PC-3 Cells Show Inhibited Cell Proliferation

**Figure 1:**
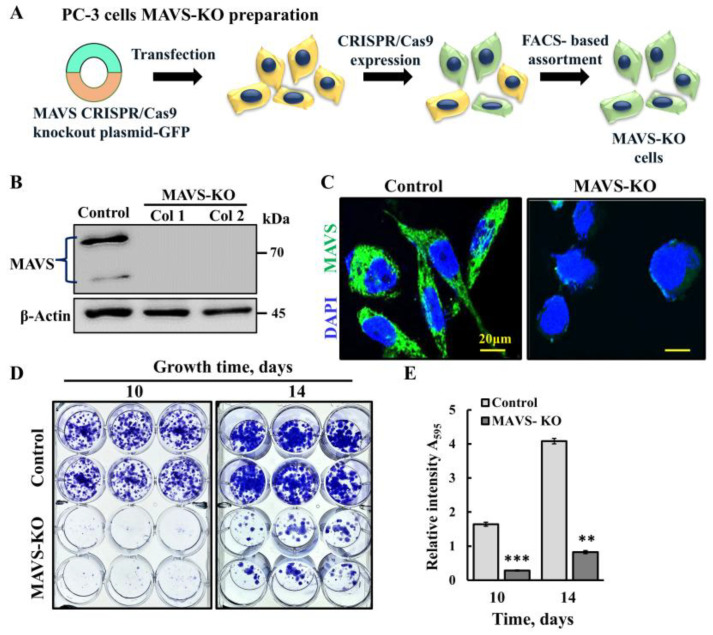
MAVS-KO in PC-3 inhibits cell proliferation. (**A**) Schematic presentation of CRISPR/Cas9 generation of MAVS-deficient (MAVS-KO) PC-3 cells. (**B**,**C**) Immunoblot (**B**) and immunofluorescence staining of MAVS (red) (**C**), representing CRISPR/Cas9-generated MAVS-KO PC-3 cells. Nuclei were counterstained with DAPI (blue). (**D**,**E**) Clonogenic assay of PC-3 cells. MAVS-expressing and MAVS-KO PC-3 cells were grown for 10 or 14 days, then stained with crystal violet (**D**), and quantified for crystal violet intensity by measuring absorbance at 595 nm (**E**). Results are the means ± SEM (*n* = 3), ** *p* ≤ 0.01; *** *p* < 0.001. Original western blots can be found at Supplementary Materials.

To further demonstrate that MAVS is a key effector in cancer progression, MAVS was depleted from prostate cancer PC-3 cells using CRISPR/Cas9-mediated MAVS knockout (MAVS-KO) (Figure 1A). As expected, these MAVS-KO cells showed no MAVS expression, as revealed using immunoblotting and immunofluorescence (IF) (Figure 1B,C). These MAVS-KO cells, assayed 10 and 14 days post-cell seeding, showed highly decreased cell proliferation. Furthermore, a clonogenic assay reflected decreased colony formation (over 80%) compared to MAVS-expressing PC-3 cells (Figure 1D,E). This result is in agreement with MAVS depletion using specific siRNA [18].

### 3.2. MAVS-KO Cells Produce Very Small Tumors with Inhibited Proliferation and Activated Apoptosis

**Figure 2:**
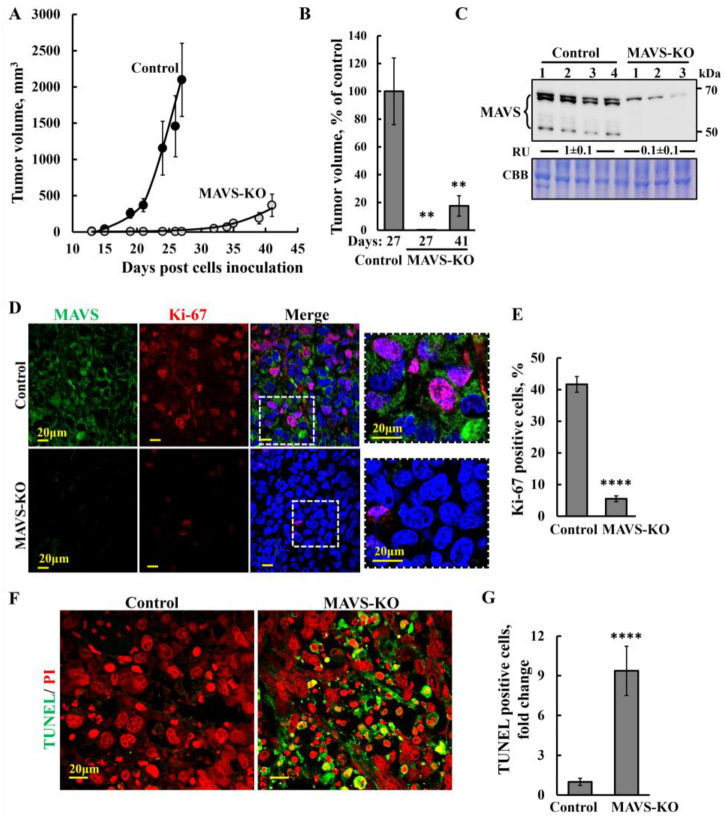
Inhibition of tumor growth and cell proliferation, and cell death induction in a MAVS-KO PC-3 prostate cancer xenograft. Control or MAVS-KO PC-3 cells (2 × 10^6^ cells/mouse) were inoculated into athymic male mice. (**A**,**B**) Tumor volumes were monitored (using a digital caliper) for 41 days, and the calculated average tumor volumes (**A**) and the relative volume on day 27 (Control) and on day 27 and 41 (MAVS-KO) (**B**) are presented. (**C**) Immunoblot analysis of MAVS in protein extracts from tumors derived from control and MAVS-KO PC-3 cells, with their relative levels presented as relative units (RUs), and Coomassie brilliant blue (CBB) staining of the samples shown as a loading control. Original western blots can be found at Supplementary Materials. (**D**,**E**) Sections of paraffin-embedded control and MAVS-KO PC-3 cell-derived tumors were IF-stained using specific anti-MAVS (green) and anti-Ki-67 (red) antibodies and their co-localization (pink), with representative confocal images (**D**) and quantification (**E**) shown. Nuclei were counterstained with DAPI (blue). (**F**,**G**) TUNEL (green) and propidium iodine (PI, red) staining, representative confocal images (**F**), and quantification (**G**) are shown. Results represent the means ± SEM (*n* = 3), ** *p* ≤ 0.01; **** *p* ≤ 0.0001.

Next, we tested the ability of MAVS-KO PC-3 cells to form tumors in nude mice. Cells expressing MAVS and lacking MAVS were injected s.c., and tumor growth was followed for 41 days. While the tumor volume of control PC-3 cells grew exponentially, reached about 2100 mm^3^ by day 27, no tumors developed in mice inoculated with MAVS-KO cells, and only by day 41 post-cell inoculation did the tumor size reach about 400 mm^3^ (Figure 2A,B). Mice weight analysis (Figure S1) indicates that, in the control group, the mice weight remained relatively stable, with a slight decrease, whereas in mice implanted with MAVS-KO cells, the weight continued to increase over time.

Immunoblot analysis of the tumors confirmed, as expected, a ~90% reduction in MAVS levels in tumors derived from MAVS-KO cells (Figure 2C). The residual MAVS signal in MAVS-KO tumors likely originates from host cells, as the antibody used detects both human and mouse MAVS.

The inhibited cell proliferation in the tumors derived from the MAVS-KO cells is represented by a decrease (over 80%) in the expression levels of the cell proliferation factor Ki-67, as shown by IF staining (Figure 2D,E).

To analyze whether MAVS depletion activates cell death, we analyzed sections derived from control and MAVS-KO tumors with TUNEL staining, detecting that DNA fragmentation occurred in the last phase of apoptosis (Figure 2F,G). While small amounts of TUNEL-positive cells were apparent in the control tumors, most of the cells in the MAVS-KO tumors were TUNEL-positive, with staining co-localizing with nuclei stained with PI (Figure 2F,G).

### 3.3. MAVS Depletion Altered the Tumor Microenvironment (TME)

**Figure 3:**
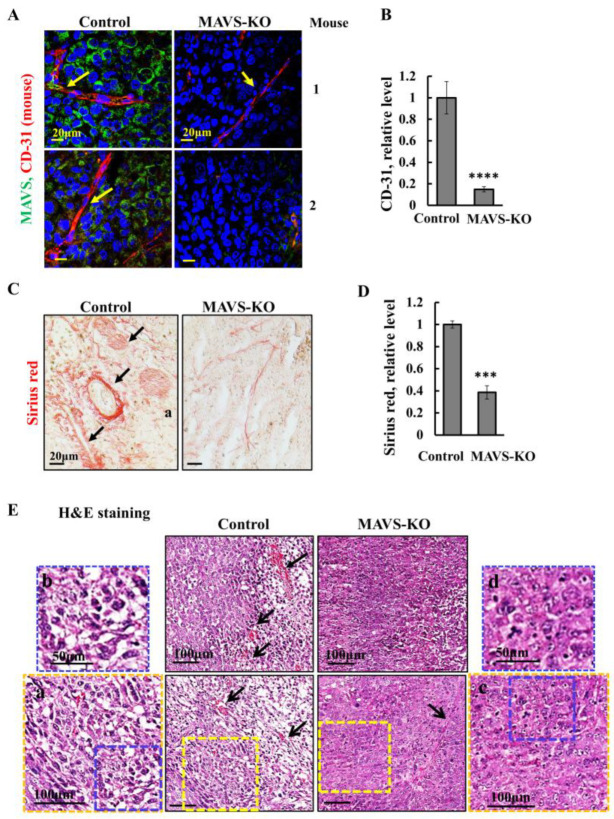
MAVS-depleted tumors show an altered tumor microenvironment and morphology. (**A**) Representative IF staining from 2 mice and quantification (**B**) of sections from control and MAVS-KO PC-3 cell-derived tumors stained with specific antibodies against MAVS (green) and CD-31 (red), with yellow arrows pointing to blood vessels. Nuclei were counterstained with DAPI (blue). (**C**) Tumor sections from control and MAVS-KO mice stained for collagen with Sirius red, a fibrosis marker. Representative images are shown, with black arrows pointing to collagen layer thickness. (**D**) Quantification of the Sirius red-stained collagen was performed using ImageJ software (version 1.54p; Bethesda, MD, USA). (**E**) Tumor sections from control and MAVS-KO mice were stained with hematoxylin and eosin, with arrows pointing to vasculature in the tumors. An enlargement of the indicated squares shows the different morphologies in control and MAVS-KO tumors (a–d). Results = mean ± SEM (*n* = 3) *** *p* < 0.001; **** *p* ≤ 0.0001.

The endothelial cell marker, CD-31, is well-established for monitoring blood vessel density in malignant tissues [33]. CD-31 staining of sections from control and MAVS-KO cell-derived tumors with specific antibodies revealed an over 80% decrease in MAVS-KO tumors relative to those expressing MAVS (Figure 3A,B), implying that the lack of MAVS affects angiogenesis.

To follow the effect of MAVS depletion on the TME, we stained for collagen-reflecting alterations [34] using Sirus Red staining. Sirus Red highly stained the collagen in MAVS-expressing cells, showing a decrease of over 60% in MAVS-KO-derived tumors (Figure 3C,D).

Hematoxylin and eosin (H&E) staining of sections from control, MAVS-expressing, and MAVS-KO tumors revealed distinct morphologies. In addition to reduced angiogenesis, MAVS-deficient tumors displayed more condensed tissue architectures and nuclei compared to MAVS-expressing tumors (Figure 3E).

### 3.4. MAVS Depletion in Prostate Cancer Increased CD-4 Tumor Infiltration and Decreased PD-L1 Expression and Cancer Stem-Cell Marker CD-44

As MAVS is a central adaptor in the antiviral innate immune response, we assessed how its loss impacts innate and adaptive immunity. In this study, we used nude mice carrying a FOXN1 mutation, which results in an athymic phenotype and deficiency of conventional αβ T cells, including Th1, Th2, Th17, CD8^+^, and Treg subsets. These mice, however, retain other immune components such as myeloid cells (macrophages, granulocytes, antigen-presenting cells), NK cells, B cells, γδ T cells, and NKT cells [35]. Despite being immunocompromised, nude mice can support extrathymic T-cell development. Several sites—including the liver, intestine, salivary glands, uterus, lymph nodes, and spleen—support T-cell maturation in mice and humans [36,37]. For instance, the liver serves as a niche for T-cell development in athymic mice [38], and a sequential maturation pathway from multipotent progenitors (CD34^+^CD38^dim^Lin^−^) to CD3^+^ T cells has been described in human tonsils [39]. Functional TCRαβ^+^ T cells have also been reported to develop extrathymically in the spleen and lymph nodes of bone marrow transplant recipients [40].

**Figure 4:**
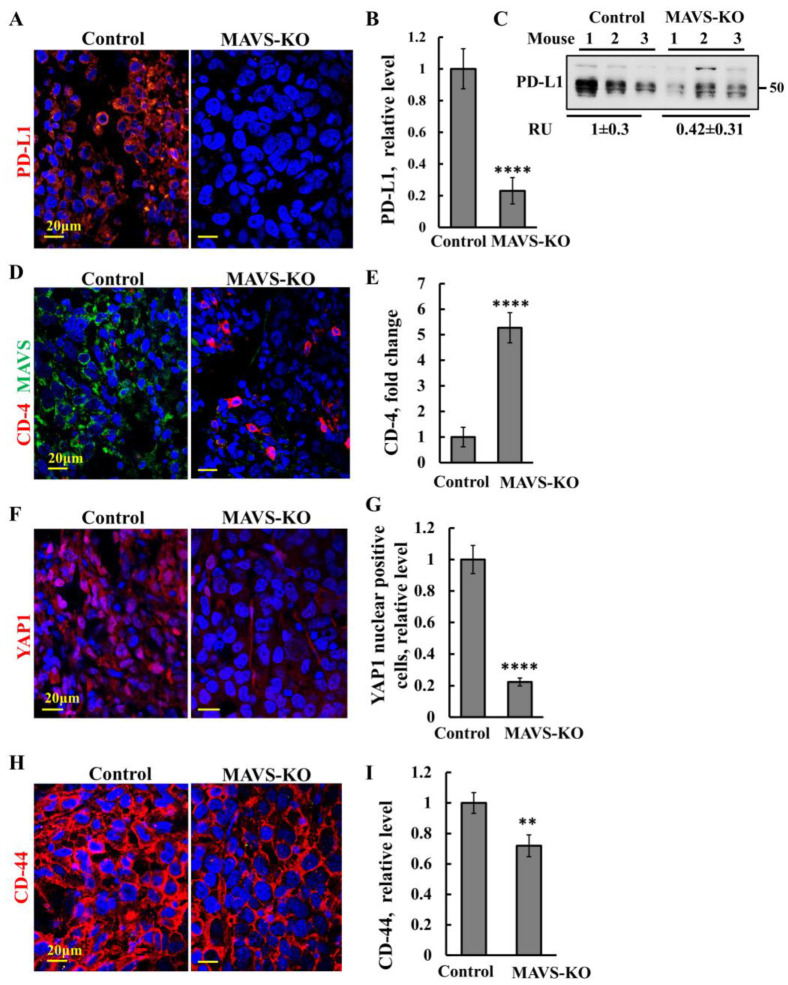
MAVS-depleted tumors show decreased PD-L1 and YAP and increased CD-4+T-cell infiltration. (**A**) Representative IF-stained sections from control and MAVS-KO PC-3 cell-derived tumors stained with human specific antibodies against PD-L1 (red) (**A**) and their quantification (**B**). (**C**) Immunoblotting of PD-L1 in proteins extracted from control and MAVS-KO-derived tumors, with their relative levels presented as relative units (RUs). Original western blots can be found at Supplementary Materials. (**D**–**I**) Representative IF staining of sections from control and MAVS-KO cells-derived tumors for MAVS (green), CD-4 (red) (**D**) and their co-localization (pink), YAP (red) (**F**) and CD-44 (red) (**H**), with their quantification (**E**,**G**,**I**), as performed using ImageJ software (version 1.54p; Bethesda, MD, USA). Nuclei were counterstained with DAPI (blue). Results represent the mean ± SEM (*n* = 3) ** *p* ≤ 0.01, **** *p* ≤ 0.0001.

As programmed cell death ligand 1 (PD-L1) plays a key role in immune evasion [41], we analyzed its expression in tumors derived from MAVS-expressing and MAVS-KO PC-3 cells using IF with specific antibodies (Figure 4A,B). Some prostate cancer cell lines, including PC-3, express PD-L1 at baseline, but strongly upregulate it in response to inflammatory cytokines such as IFN-γ, consistent with adaptive immune resistance [42]. Consistent with this, our results show that PD-L1 levels, detected with an antibody recognizing the human protein, were markedly reduced (~80%) in tumors derived from MAVS-KO PC-3 cells (Figure 4A,B). Immunoblot analysis of tumor extracts further confirmed this substantial decrease in PD-L1 in MAVS-depleted tumors (Figure 4C).

Next, we tested tumor sections from control and MAVS-KO cells for the levels of CD-4+T cells using specific antibodies (Figure 4D,E). Tumors derived from MAVS-KO cells showed five-fold higher CD-4+T cell infiltration (Figure 4D,E(. Thus, MAVS had clearly altered the TME and made it prone to immune eradication, rather than immune evasion. Yes-associated protein (YAP1) is a transcription factor for *PD-L1* [43], promoting cancer stem-cell (CSC) formation [44]. Therefore, we compared the expression levels of YAP1 in tumor sections from control and MAVS-depleted tumors (Figure 4F,G). We found that the YAP1 expression level was significantly decreased (70%) in the MAVS-KO-derived tumors (Figure 4F,G), along with its nuclear translocation (Figure 4F,G).

To follow the effect of MAVS depletion on cancer stem-cell levels in the PC-3 cell-derived tumors, we analyzed the levels of the prostate cancer stem-cell marker CD-44 [45], which played a critical role in tumor progression [46,47]. An IF staining analysis with specific antibodies of CD-44 expression showed a high decrease in the CD-44 levels in the MAVS-KO tumors compared to the MAVS-expressing tumors (Figure 4H,I), suggesting that MAVS plays a role in CSC differentiation.

### 3.5. Mass Spectrometry Analysis of the Differentially Abundant Proteins in MAVS-KO Cells

**Figure 5:**
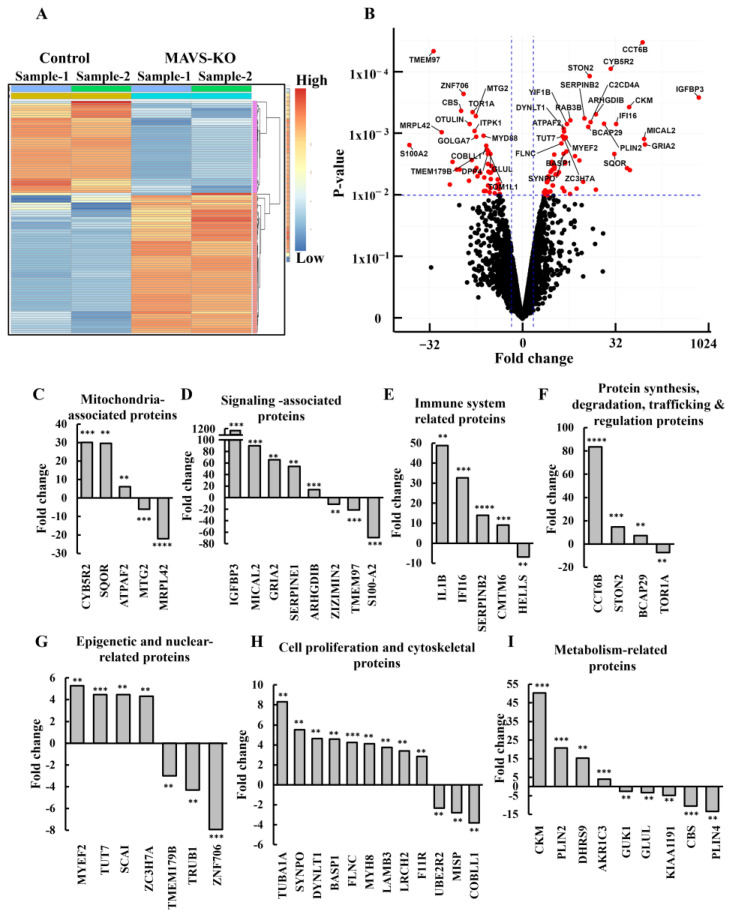
Differentially abundant proteins in MAVS-KO- and MAVS-expressing cells. Human proteins differentially expressed between MAVS-expressing and MAVS-depleted PC-3 cells were analyzed by LC-HR-MS/MS. Human proteins presenting at least one unique peptide, those with a nominal *p*-value smaller than 0.01, and linear fold change higher than 1.5 in either direction are presented. (**A**) Hierarchical clustering of the 96 proteins found to be differentially expressed between MAVS-expressing and MAVS-depleted cells. The color scale of the standardized expression values is shown. (**B**) Volcano plot shows *p*-values as a function of the fold change in MAVS-KO cells relative to MAVS-expressing cells. Red circles represent significantly differentially expressed proteins, while black circles represent proteins with non-significant changes. (**C**–**I**) Differential expressions of proteins in MAVS-KO relative to MAVS-expressing PC-3 cells were quantitatively analyzed using LC-HR MS/MS data**.** These include proteins related to (**C**) mitochondria, (**D**) signaling, (**E**) immune system, (**F**) protein synthesis, degradation, trafficking, and regulation, (**G**) epigenetic and nuclear factors, (**H**) cell proliferation and cytoskeleton, and (**I**) metabolism. Results = mean ± SEM, ** *p* ≤ 0.01, *** *p* ≤ 0.001, **** *p* ≤ 0.0001.

To identify the proteins showing different expression levels in MAVS-KO PC-3 cells and their association with inhibited cancer-cell proliferation and altered TME, inflammation, and immunity, CRISPR/Cas9 MAVS-depleted cells and cells expressing MAVS were subjected to proteomics profiling (LC-HR MS/MS), and differentially abundant proteins were grouped into functional categories (Figure 5, Tables S2–S8). An analysis for human proteins that had at least one unique peptide and were expressed in at least one of the biological groups identified 4638 proteins, which were submitted for subsequent analysis. The differentially expressed proteins [*p*-value < 0.01 and fold change (FC) ≥ 1.5 in either direction] between cells expressing or depleted of MAVS were 96, of which 58 proteins were upregulated and 38 were downregulated. As expected, MAVS was highly reduced in MAVS-KO cells (Table S2). The hierarchical clustering of the differently expressed proteins in cells expressing or depleted of MAVS (Figure 5A) and the volcano plot (Figure 5B) show that larger numbers of the differentially expressed proteins were upregulated upon MAVS depletion than were downregulated.

The human proteins differentially expressed upon MAVS depletion in PC-3 human cancer cells were sub-grouped according to cellular functions and are presented along with their subcellular localization (Tables S2–S8). These include proteins associated with the mitochondria (Figure 5C, Table S2), signaling-related proteins (Figure 5D, Table S3), immune system-related proteins (Figure 5E, Table S4), protein synthesis, degradation, trafficking and regulation (Figure 5F, Table S5), epigenetic- and nuclear-related proteins (Figure 5G, Table S6), cell proliferation and cytoskeletal proteins (Figure 5H, Table S7), and metabolism-related proteins (Figure 5I, Table S8). The relation of these proteins to various diseases is also indicated.

Mitochondria-associated proteins whose expression was altered in the MAVS-KO PC-3 cells (Table S2) include several proteins with markedly increased levels, such as NADH-cytochrome b5 reductase 2 (CYB5R2; +30-fold), which is involved in fatty acid desaturation, cholesterol biosynthesis, and drug metabolism, sulfide:quinone oxidoreductase (SQOR; +29.6-fold), catalyzes the oxidation of hydrogen sulfide to thiosulfate and the ATP Synthase F1 complex assembly factor 2 (ATPAF2; +6-fold), and plays a crucial role in assembly of the F1 subunit of ATP synthase.

The proteins that were highly decreased (Table S2) include the 39S ribosomal protein L42 (MRPL42), which is involved in protein synthesis in the mitochondria and decreased 22-fold; the mitochondrial ribosome-associated GTPase 2 (MTG2; −6-fold), which regulates the mitochondrial ribosome assembly and translational activity.

Among the 20 signaling-related proteins whose expression was altered in the MAVS-KO cells (Table S3, Figure 5D) are the insulin-like growth factor-binding protein 3 (IGFBP3), whose levels were highly increased (988-fold). This protein is the main insulin growth factor (IGF) transport protein in the bloodstream that promotes the effects of (IGF), and it is highly effective as a pro-apoptotic factor in tumor cells (Table S3). F-actin-monooxygenase (MICAL2), which promotes depolymerization of F-actin and controls proliferation and migration of cancer cells, was also highly upregulated (90-fold). Similarly, the plasminogen activator inhibitor 1 (SERPINE1), an inhibitor of tissue-type plasminogen activator (PLAT) and urokinase-type plasminogen activator, increased +54.28-fold (Table S3).

The proteins S100A2 and S100A6 (Table S3), which function as a calcium sensor and modulate Ca^2+^ signaling, were significantly decreased (−69- and −5.32-fold, respectively) in the MAVS-KO prostate cancer cells. Sigma intracellular receptor 2 (TMEM97), an intracellular orphan receptor that is highly expressed in proliferating cancer cells (Table S3), was highly downregulated (−21-fold) in the MAVS-KO cells. Other downregulated proteins in the signaling proteins included RANBP2-type and C3HC4-type zinc finger containing 1, and the dedicator of cytokinesis protein 11 (DOCK11), which all decreased by about ten-fold (Table S3).

The expression of nine immune system-related proteins was altered in the MAVS-KO cells (Figure 5E, Table S4). These included proteins with increased expression such as the IL-1β (+48.8-fold), a key pro-inflammatory cytokine involved in innate immune response, and the gamma-interferon-inducible protein 16 (IFI16) (32.6-fold). IFI16 binds to double-stranded supercoiled DNA and is involved in the innate immune response by recognizing viral dsDNA in the cytosol and in the nucleus, leading to the induction of IFN-β and other interferon-related cytokines (Table S4). Plasminogen activator inhibitor 2 (SERPINB2) increased 13.9-fold. SERPINB2 is serin protease inhibitor, involved in inflammation, immune responses, and regulation of plasminogen activation (Table S4). Chemokine-like factor (CKLF)-like MARVEL transmembrane domain 6 (CMTM6) increased 9.3-fold. CMTM6 is a master regulator of expression and recycles PDL-1, an immune inhibitory ligand for immune tolerance (Table S4).

Other proteins in the immune system-related protein category that were downregulated in MAVS-KO PC-3 cells include myeloid differentiation primary response protein (MYD88; −5.56-fold) and an adaptor protein involved in Toll-like receptor and IL-1 receptor signaling pathways in the innate immune response (Table S4).

The expression of 13 proteins related to protein synthesis, degradation, trafficking, and their regulation was also altered in MAVS-KO cells (Figure 5F, Table S5). These include the molecular chaperone complex T-complex protein 1 subunit zeta-2 (CCT6B), whose level was increased 83.5-fold, and the Torsin-1A (TOR1A) whose level decreased 7.3-fold, and the B-cell receptor-associated protein 29 (BCAP29), which is an integral membrane protein of the ER that is involved in quality control and sorting and whose level increased 7.3-fold (Table S5).

The expression levels of 14 epigenetic and nuclear-related proteins were changed, and, as expected, were located mainly in the nucleus (Figure 5G, Table S6). These include the myelin expression factor 2 (MYEF2; +5.3-fold), a transcriptional repressor that mainly inhibits the transcription of the myelin basic protein gene (MBP).

The proteins that showed decreased expression include zinc finger protein 706 (ZNF706; −7.9-fold), DNA-binding transcription factors (TFs) interacting with and regulating RNA-associated processes, and pseudouridylate synthase (TRUB1), which plays a role in RNA modification (Table S6).

In the cell proliferation and cytoskeletal -17-related proteins (Figure 5H, Table S7), the upregulated proteins in the MAVS-KO cells included the tubulin alpha-1A chain (TUBA1A; +8.3-fold), a component of microtubules that maintains cell shape, enables intracellular transport, and segregates chromosomes during cell division, cell adhesion, and cell movement (Table S7); and synaptopodin (SYNPO; +5.5-fold), an actin-associated protein encoded by the SYNPO gene. It plays a crucial role in modulating the actin cytoskeleton dynein light chain tctex-type 1 (DYNLT1; +4.6-fold), a component of the cytoplasmic dynein motor complex.

The downregulated proteins included cordon-bleu protein-like 1 (COBLL1; −3.8-fold), which is involved in actin cytoskeleton remodeling and pathological processes; the mitotic interactor and substrate of PLK1 (MISP, mitotic spindle positioning) (−2.8-fold), an actin-binding protein integral to mitotic spindle orientation and cell division; and the ubiquitin-conjugating enzyme E2 R2 (UBE2R2; −2.3-fold), which inhibits protein phosphatase 2A (PP2A) during mitosis (Table S7).

Metabolism-related proteins (Figure 5I, Table S8) whose expression was modified in MAVS-KO cells include 15 proteins; those involved in cellular energy homeostasis such as creatine kinase M-type (CKM; +50-fold), which is vital; dehydrogenase, such as dehydrogenase/reductase SDR family member 9 (DHRS9; +15.4-fold) and aldo-keto reductase family 1 member C3 (AKR1C3; +4-fold); and glutamine synthetase (GLUL; −3.3-fold) and cystathionine beta-synthase (CBS, −10.54-fold). Other proteins are associated with nucleotides, such as guanylate kinase (GUK1; −2.56-fold), and steroid hormone metabolism, aldo-keto reductase family 1 member C1 (AKR1C1; +3.6-fold).

### 3.6. MAVS Depletion Alerts Immune Signaling

**Figure 6:**
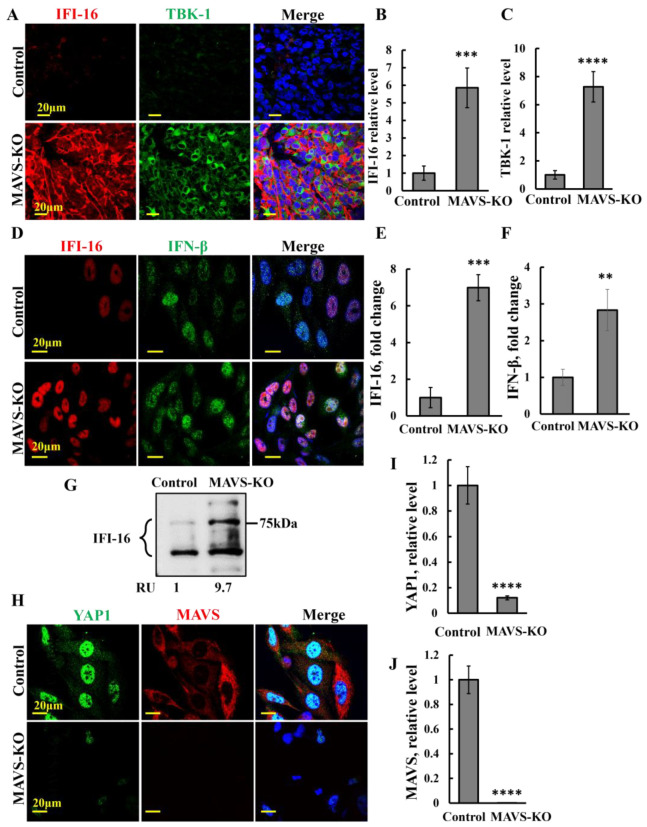
MAVS-depleted tumors show increased expression levels of IFI-16 and TBK-1, and MAVS depletion in PC-3 cells increases the expression levels of IFI-16 and IFN-β and inhibits YAP1 expression and nuclear translocation. (**A**–**C**) Representative IF staining and quantification of sections from control and MAVS-KO PC-3 cell-derived tumors co-stained with specific antibodies against IFI-16 (red) and TBK-1 (green), with nuclei stained with DAPI (blue). Representative IF images (**A**) and staining quantification (**B**,**C**) performed using ImageJ software (version 1.54p; Bethesda, MD, USA). (**D**–**F**) Representative IF staining of MAVS-KO and MAVS-expressing PC-3 cells co-stained for IFI-16 (red) and IFN-β (green) using specific antibodies, and nuclei stained with DAPI (blue) (**D**). Quantitative analysis of IFI-16 and IFN-β staining intensity (**E**,**F**). (**G**) Immunoblot of IFI-16 in MAVS-expressing (control) and MAVS-KO cells, with their relative levels presented as relative units (RUs). Original western blots can be found at Supplementary Materials. (**H**–**J**) Representative IF staining of control and MAVS-KO PC-3 cells co-stained for YAP1 (green) and MAVS (red), with nuclei stained with DAPI (blue) (**H**). Quantitative analysis of YAP1 (**I**) and MAVS (**J**) staining intensity. Results = mean ± SEM (*n* = 3), ** *p* ≤ 0.01; *** *p* ≤ 0.001; **** *p* ≤ 0.0001.

Next, from the proteomics data, we selected one of the immune system-related proteins, IFI-16, that was highly upregulated (32.6-fold) (Table S4, Figure 5E) and analyzed its expression levels using IF staining and immunoblotting, both in PC-3 cells in culture and in xenograft tumors expressing and depleted of MAVS (Figure 6A,B,D,E,G,I).

IFI16 recognizes double-stranded DNA in the cytosol and in the nucleus, leading to the induction of IFN-β and other interferon-related cytokines, and it is involved in the innate immune response. The expression of IFI-16 is reported to strongly indicate tumor suppression. Reactivation of IFI-16 genetically elicited a potent immune cytotoxicity and memory response that led to durable tumor suppression.

IF analysis using specific antibodies of the levels of IFI-16 in tumors from control and MAVS-KO PC-3 cells revealed a six-fold increase in IFI-16 levels in the MAVS-depleted tumors (Figure 6A,B,D,E), as expected, and is coherent with the proteomics analysis of MAVS-KO and MAVS-expressing PC-3 cells (Table S4, Figure 5E).

Since IFI-16 mediates TBK-1-dependent IFN-β production via an adaptor STING and it is essential for pathogens and damaged cell eradication [48], we also checked the expression levels of TBK-1 in tumors expressing and depleted of MAVS (Figure 6A,C). The results show elevated levels (seven-fold) of TBK-1 in the MAVS-KO-derived tumors.

In the MAVS-KO cells in culture, IFI-16 expression levels, as analyzed by IF, increased over 18-fold (Figure 6D,E). In the MAVS-KO cells, immunoblotting demonstrated an increase in IFI16 levels that appeared as several protein bands (Figure 6G).

As IFI-16 mediates TBK-1-dependent IFN-β production via an adaptor STING and is essential for pathogens and damaged cell eradication [48], and MAVS is also involved in IRF-3 induced IFN-β production during viral infection [48], we also examined the effect of MAVS depletion on IFN-β expression (Figure 6D,F). The levels of IFN-β also increased (three-fold) in the MAVS-KO cells.

IFI16 is a major activator of IRF3, which facilitates YAP1 nuclear translocation. Additionally, as we demonstrated previously, there was a significant decline in YAP1 nuclear translocation in si-RNA MAVS-treated cells [18]; thus, we analyzed YAP expression levels in MAVS-KO cells (Figure 6H,I). YAP1 is a tumorigenic protein that is pivotal in tumor proliferation. We found that the levels of YAP1 were highly decreased (90%) in the MAVS-KO cells. As expected, the level of MAVS in these cells was close to zero (Figure 6H,J).

## 4. Discussion

Globally, prostate cancer is one of the leading causes of cancer morbidity and mortality, ranking as the second most frequent malignancy in males, with an estimated 1.4 million diagnoses and 375,000 deaths worldwide in 2020.

**Figure 7:**
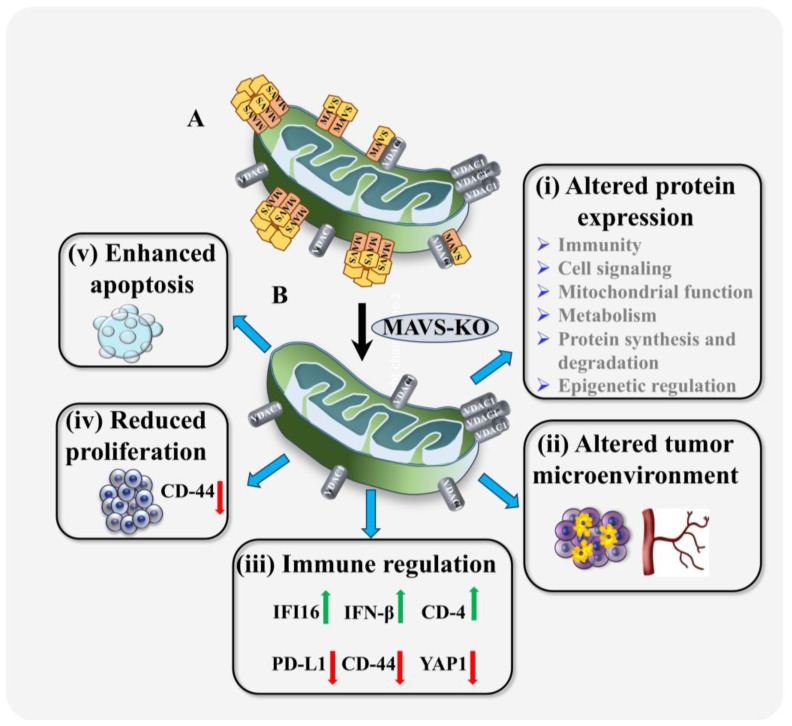
Proposed multifunctional roles of MAVS in prostate cancer revealed by MAVS-KO: implications for a multi-target therapeutic strategy. Schematic representation of mitochondrial MAVS function in prostate cancer cells and the consequences of MAVS depletion. (**A**) In prostate cancer cells, MAVS is overexpressed and forms oligomeric complexes, a fraction of which is associated with VDAC1 on the mitochondrial outer membrane. MAVS regulates and maintains multiple cellular functions, including innate antiviral immunity, inflammatory cytokine signaling, cell proliferation, and diverse physiological and metabolic processes essential for cancer cell survival. (**B**) Knockout of MAVS (MAVS-KO) leads to broad cellular and tumor-level changes, including: (i) altered expression of proteins involved in immunity, cell signaling, mitochondrial function, metabolism, protein synthesis and degradation, and epigenetic regulation; (ii) remodeling of the tumor microenvironment, reflected by reduced angiogenesis and extracellular matrix expression; (iii) modulation of the immune response, characterized by increased tumor infiltration of CD-4^+^ cells and elevated expression of IFN-β and IFI-16 (green arrows), alongside decreased infiltration of CD-44^+^ cells, representing cancer stem-cells and reduced expression of PD-L1 and YAP1 (red arrows); (iv) reduced cancer cell proliferation; and (v) induction of apoptosis. Collectively, these findings indicate that MAVS exerts broad pro-tumorigenic effects through modulation of interconnected protein networks and signaling pathways and that its loss promotes tumor-suppressive outcomes.

In this study, prostate cancer PC-3 cells were knocked out for MAVS expression via CRISPR/CAS9 and were studied in cells in culture and in tumor xenografts derived from these cells, with respect to the multifunction of MAVS. The results obtained including a proteomic analysis reveal that MAVS functions not only as an anti-viral protein, but its depletion alters the expression of proteins related to immunity, signaling, cell proliferation, cytoskeletal and mitochondrial function, metabolism epigenetics, and protein synthesis, degradation, and regulation (Figure 7). The association of MAVS with the above cell functions can explain the findings that tumor development of the MAVS-KO cells was highly decreased, showing inhibited cell proliferation, CSC elimination, induced cell death, and altered (TME) and immune response, making it more susceptible to immune eradication, rather than immune evasion. Similar findings were recently demonstrated upon MAVS depletion in cancer cells using specific si-RNA [18].

### 4.1. MAVS Depletion Reduces Proliferation, Induces Cell Death, and Alters the Tumor Microenvironment

Consistent with earlier findings using MAVS-specific siRNA, MAVS depletion in PC-3 cells significantly reduced proliferation [18]. In xenograft tumors derived from MAVS-KO cells, growth was markedly suppressed, as evidenced by reduced expression of the proliferation marker Ki-67 (Figure 2D,E). Tumors lacking MAVS underwent extensive cell death, as revealed by TUNEL assay (Figure 2F,G and Figure 7).

The decreased CD-44 levels observed in our model are consistent with reduced tumor aggressiveness (Figure 7). CD-44 is a well-established CSC marker and is associated with tumor cell plasticity, invasion, metastasis, and immune evasion. Elevated CD-44 expression has been linked to therapeutic resistance and poor prognosis in prostate cancer and other malignancies. Accordingly, reduced CD-44 expression suggests a loss of stemness-like tumor cell populations and attenuation of epithelial–mesenchymal transition (EMT). These changes are concordant with the observed reduction in cell proliferation, increased apoptosis, and decreased YAP1 signaling. Importantly, downregulation of CD-44 may also facilitate immune cell infiltration by weakening tumor–stroma interactions and immune-exclusion mechanisms. Thus, the combined increase in CD-4^+^ T-cell infiltration and reduction in CD-44 expression supports the presence of a less aggressive and more immunologically permissive tumor microenvironment characterized by diminished tumor stemness and enhanced antitumor immune surveillance.

MAVS depletion further influenced tumor microenvironment-associated factors. Angiogenesis, a well-established hallmark of cancer, was impaired. CD-31, a prominent angiogenesis marker, was highly reduced in MAVS-KO PC-3 cell-derived tumors compared to control (Figure 3A,B). Collagen deposition, typically elevated in malignant and metastatic tumors, was also diminished in MAVS-KO tumors, as shown by Sirius Red staining (Figure 3C,D). Histological analysis revealed distinct morphological differences: MAVS-expressing tumors displayed vesiculation and multinucleated cells, features largely absent in MAVS-depleted tumors (Figure 3E).

Together, these findings suggest that MAVS is critical for sustaining tumor proliferation, survival, and shaping the tumor microenvironment.

### 4.2. MAVS Depletion Reshapes Tumor Immunity Through Altered Protein Expression

Tumors derived from MAVS-deficient PC-3 cells exhibit marked alterations in immune-related pathways (Figure 4, Figure 5 and Figure 6). Specifically, MAVS-KO tumors show a pronounced reduction in PD-L1 and YAP1 expression, accompanied by increased CD-4^+^ T-cell infiltration and elevated levels of IFI-16 (Table S4, Figure 5E).

MAVS depletion significantly enhanced intratumoral CD-4^+^ T-cell infiltration (Figure 4D,E), a feature associated with improved prognosis and predictive of responsiveness to immune checkpoint inhibitors (ICIs) across multiple cancer types [49]. CD-4^+^ T cells are central mediators of antitumor immunity, and their increased presence reflects enhanced immune surveillance and a shift toward a more immunostimulatory TME [50]. Such immune activation is often linked to changes in immune checkpoint signaling and activation of interferon pathways, consistent with the elevated IFN-β levels observed in MAVS-depleted tumors. These responses have been associated with improved outcomes and clinical responsiveness to anti–PD-1 therapy in cancers such as non-small cell lung cancer (NSCLC) and bladder cancer [51,52]. Thus, the increase in CD-4^+^ T cell tumor infiltration upon MAVS depletion may provide avenues for therapeutic intervention.

Consistent with this immune-activating phenotype, YAP1—a transcriptional regulator of *PD-L1* [43]—was markedly reduced in MAVS-KO tumors (Figure 4F,G), in agreement with our previous siRNA-based findings [18]. This supports a model in which MAVS promotes immune tolerance through the YAP1–PD-L1 axis.

PD-L1 plays a central role in tumor immune evasion by suppressing T-cell proliferation and activity through the engagement of PD-1. Elevated PD-L1 expression has been linked to poor prognosis and serves as a predictive biomarker for response to PD-1/PD-L1-targeted therapies.

In prostate cancer, PD-L1—but not PD-1—is highly prevalent, correlates with Gleason score, and is associated with disease progression and aggressiveness [53]. Additionally, higher PD-L1 expression in prostate cancer is linked to a more aggressive disease state [54,55,56,57]. Therefore, the reduction in PD-L1 expression upon MAVS depletion is likely to enhance immune-mediated tumor clearance.

Proteomic and immunostaining analyses further revealed an increased expression of IFI-16 and its downstream effector IFN-β in MAVS-deficient tumors (Figure 5E and Figure 6). IFI-16 functions as a key link between innate and adaptive immunity [58], has been associated with improved responsiveness to ICIs in melanoma [59], and suppresses colony formation in prostate cancer cells [60]. Thus, elevated IFI-16 expression following MAVS loss is expected to further potentiate antitumor immune responses and enhance sensitivity to immunotherapy.

Collectively, these findings demonstrate that MAVS plays a critical role in maintaining tumor immune tolerance. Its depletion disrupts immune suppressive mechanisms, promotes antitumor immunity, and may create new opportunities for enhancing immunotherapeutic strategies.

### 4.3. Proteomics Analysis of MAVS-KO Cells Revealed Global Changes in Cell Functions

A proteomic analysis provided insight into altered protein expression and cellular functions following MAVS depletion. An LC-HR-MS/MS analysis of prostate cancer PC-3 cells expressing and depleted of MAVS revealed 96 differentially expressed proteins, including 58 that were upregulated and 38 that were downregulated. These differentially expressed proteins point to alterations in mitochondrial proteins, signaling pathways, immunity, metabolism, proliferation, the cytoskeleton, epigenetics, protein synthesis, degradation, trafficking, and regulation.

The expression of the mitochondria-associated proteins in MAVS-KO PC-3 cells were strongly altered (Figure 5C, Table S2). In addition to MAVS loss, the 39S ribosomal protein L42 (MRPL42), essential for mitochondrial protein synthesis, decreased ~22-fold. Conversely, sulfide:quinone oxidoreductase (SQOR; +29-fold) was upregulated, indicating a broad disruption in mitochondrial metabolism, protein synthesis, and assembly.

The proteins associated with cell signaling (Figure 5D, Table S3) included a striking 988-fold increase in insulin-like growth factor-binding protein 3 (IGFBP3), which is a pro-apoptotic and anti-proliferative regulator in tumorigenic cells [61]. In the nucleus, IGFBP3 modulates nuclear hormone receptor activity by directly binding to receptors such as the retinoid X receptor, retinoic acid receptor [62], and PPARγ [63]. It also interacts with DNA-dependent protein kinase to promote DNA repair [64].

IGFBP3 exerts anti-proliferative effects in many cell types by blocking the ability of IGF-1 and IGF-2 to activate IGF1R. It acts as a low-penetrance tumor suppressor gene, and its dysregulation is implicated in many cancers. Elevated IGFBP3 within tumors is linked to increased cancer severity [65], while its expression decreases during prostate cancer progression from benign to metastatic disease [66]. Thus, its upregulation in MAVS-KO cells may represent an anti-cancer effect.

In contrast, S100A2, a Ca^2+^ sensor with context-dependent tumor roles, was downregulated (−69-fold). Interestingly, its expression is reduced in lung, kidney, prostate cancer, and melanoma [67], but in some cases, it has been reported as being overproduced [68]. TMEM97 (σ2R2/MAC30), a member of the insulin-like growth factor-binding protein family, was markedly reduced (−21-fold). Normally, it is highly expressed in proliferative cells and regulates Ca^2+^ and cholesterol trafficking (Table S3). It is differentially expressed across tumor types, where it influences cell proliferation, differentiation, and apoptosis [69]. Its expression is elevated in breast, colon, gastric, esophageal, lung, ovarian, and prostate cancers, but reduced in pancreatic and renal cancers [70].

High TMEM97 expression in proliferating cancer cells makes it a diagnostic and therapeutic target [70]. Its strong TMEM97 downregulation in MAVS-KO cells is consistent with reduced proliferation (Figure 1D,E and Figure 2D,E) and a decrease in cell-cycle proteins (Figure 5H, Table S7). This marked reduction indicates MAVS downregulation to be a potential therapeutic strategy.

MAVS depletion also highly impacts immune protein expression (Figure 5E, Table S4), where most of the proteins playing a vital role in eliciting the immune system were upregulated upon MAVS depletion. For example, IL-1β, a key pro-inflammatory cytokine involved in innate immune response, highly increased (+48.8-fold), as well as IFI-16 (+32.6-fold), a ds-DNA and RNA recognizing protein that is found in the cytosol and nucleus. It prompts an innate immune response by inducing IFN-β, along with other interferon-related cytokines, in viral infections.

CMTM6 is a master regulator of PD-L1 expression in cancer cells, recycling this immune-inhibitory ligand to promote immune tolerance [71]. PC-3 cells express PD-L1; they generally also express the PD-L1 regulator CMTM6 [72].

As discussed above, PD-L1 overexpression mediates tumor immune escape, while a PD-1/PD-L1 blockade enhances anti-tumor immunity and survival. As shown here, MAVS depletion in tumors reduced PD-L1 expression by 75% (Figure 4A–C), but increased CMTM6 expression nine-fold (Table S4).

Since CMTM6 stabilizes PD-L1 and prevents its lysosomal degradation, this inverse relationship of increased CMTM6 and decreased PD-L1 is unexpected. Given that CMTM4, a close homolog, can act as a tumor suppressor in renal and colorectal cancers and shares many functions with CMTM6 [73], it is possible that in prostate cancer, CMTM6 may likewise serve a tumor-suppressive role (Table S4).

Although CMTM6 usually stabilizes PD-L1, its function may be influenced by cellular context. In prostate cancer, post-translational modifications, interactions with other proteins or stress pathways may alter its effect, allowing for PD-L1 degradation despite CMTM6 upregulation. Additionally, cells may upregulate CMTM6 in response to decreased PD-L1 as a compensatory attempt to restore immune evasion. Increased CMTM6 may not be localized to site of PD-L1 trafficking occurs; for example, it may be predominantly present in intracellular compartments rather than at the plasma membrane, thereby limiting its stabilizing effect. Thus, while CMTM6 stabilizes PD-L1 under normal conditions, MAVS depletion likely disrupts upstream signals (e.g., interferon-mediated transcription or trafficking pathways), overriding CMTM6’s stabilizing role and leading to reduced PD-L1, despite elevated CMTM6.

Other immune-related proteins downregulated in MAVS-KO prostate cancer cells (Table S4) include the myeloid differentiation primary response protein (MYD88), an adaptor in Toll-like and IL-1 receptor signaling of innate immunity.

The expression of TRAF-type zinc finger domain-containing protein 1 (TRAFD1), which controls excessive innate immune responses, was also reduced (−3.9-fold). These findings suggest that MAVS plays a role in immune activation in cancer and can modulate tumor–immune interactions.

MAVS-KO in PC-3 prostate cancer cells also altered proteins involved in synthesis, degradation, and trafficking pathways (Figure 5F, Table S5). These include complex protein 1 subunit zeta-2 (CCT6B), which increased (+83-fold); this is part of a chaperone complex that ensures that the proper folding of proteins, such as actin, tubulin, and other cytoskeletal or cell-cycle proteins. STON2 (+14.6-fold), involved in synaptic vesicle recycling is elevated. 

Epigenetic- and nuclear-related proteins (Figure 5G, Table S6) upregulated in MAVS-KO cells include a histone chaperone required for chromatin refolding. The tumor suppressor SCAI showed context-dependent effects: high expression correlates with better survival in breast and lung cancers, but poorer outcomes in gastric, prostate, and colorectal cancers (Figure 5G, Table S6).

Myelin expression factor 2 (MYEF2; +5.2-fold), a transcriptional repressor promoting migration and invasion in hepatocellular carcinoma, yet high MYEF2 in glioblastoma multiforme (GBM), has been linked to better outcomes and lower malignancy (Figure 5G, Table S6).

Downregulated proteins include zinc finger protein 706 (ZNF706; −7.96-fold), a DNA-binding transcription factor regulating RNA-associated processes, whose high expression is associated with poor hepatocellular carcinoma (HCC) prognosis. These results indicate that MAVS contributes to epigenetic regulation in the tumorigenesis of PC-3 cells.

In MAVS-KO cells, cell proliferation- and cytoskeleton-related proteins (Figure 5H, Table S7) showed notable changes: upregulated tubulin alpha-1A chain (TUBA1A; +8.3-fold), critical for cell shape, transport, chromosome segregation, adhesion, and movement; upregulated synaptopodin (SYNPO; +5.5-fold), an actin cytoskeleton regulator; and upregulated dynein light chain Tctex-type 1 (DYNLT1; +4.6-fold), part of the dynein motor complex. Downregulated proteins include cordon-bleu protein-like 1 (COBLL1; −3.8-fold), involved in actin remodeling; mitotic interactor and substrate of PLK1 (MISP; −2.8-fold), essential for spindle orientation and division; and ubiquitin-conjugating enzyme E2 R2 (UBE2R2, −2.3-fold), an inhibitor of PP2A during mitosis (Figure 5H, Table S7). These changes collectively affect microtubule and actin dynamics, as well as mitotic control.

The metabolism-related proteins (Figure 5I, Table S8) altered include those in energy homeostasis, such as creatine kinase M-type (CKM; +50-fold); Perilipin-2 (PLIN2; +20-fold), a regulator of lipid turnover; dehydrogenases SDR family member 9 (DHRS9; +15.4-fold); and aldo-keto reductase family 1 member C3 (AKR1C3; +4-fold). Also, glutamine synthetase (GLUL; −3.3-fold) links nitrogen metabolism, amino acid synthesis, and cellular energy balance, making it essential in both normal and cancerous cells. Others are linked to nucleotide metabolism (guanylate kinase (GUK1), −2.56-fold) and steroid hormone metabolism (aldo-keto reductase family 1 member C1 (AKR1C1), +3.6-fold).

Thus, in MAVS-KO cells, key metabolism-related proteins affect energy balance and metabolic pathways.

### 4.4. MAVS in Tumor Biology

MAVS has recently been implicated in tumor biology. Increasing evidence indicates that MAVS is overexpressed in several cancers and promotes cancer cell proliferation and inflammatory signaling [18]. Several mechanisms have been proposed through which MAVS may support tumor growth, including the promotion of chronic inflammation, immune evasion, and resistance to therapy. Chronic inflammation is a well-known driver of tumor progression, and MAVS has been shown to regulate activation of the NLRP3 inflammasome, thereby contributing to inflammatory cytokine production [74]. These processes may facilitate tumor development and immune evasion.

MAVS signaling can also suppress anti-tumor immune responses. Recent studies have highlighted MAVS as an important regulator of inflammatory and immune signaling in cancer [20]. In addition, noncanonical MAVS signaling has been shown to restrain dendritic cell-driven anti-tumor immunity by inhibiting IL-12 production, thereby limiting effective T-cell responses against tumors [75].

MAVS may exert tumor-suppressive functions under certain conditions. It has been demonstrated that MAVS can stabilize p53, promoting p53 activation and protecting against tumorigenesis [76].

Taken together, these findings, along with our observation that MAVS silencing [18] or MAVS knockout (this study) reduces tumor development, suggest that MAVS can promote tumor growth in specific cellular contexts and may represent a potential therapeutic target.

Overall, MAVS-KO cells displayed widespread proteomic remodeling, disrupting mitochondrial metabolism, cytoskeletal dynamics, mitotic control, immune regulation, and epigenetic processes, collectively reducing tumor proliferation and enhancing immune susceptibility. Furthermore, alteration in the expression of most of these is associated with diseases of many types of cancer (chronic lymphocytic leukemia; lung, prostate, breast, colon, and pancreatic cancers; melanoma; and renal cell and hepatocellular carcinomas), as well as non-malignant diseases such as muscular dystrophy and hypertrophic cardiomyopathy (Tables S2–S8).

While the present findings highlight the biological and potential therapeutic relevance of MAVS, several limitations should be acknowledged. Although CRISPR knockout can be used to eliminate a gene, it has several limitations. In the current study, the observed effects are similar to those reported with MAVS-targeting siRNA [18], suggesting that siRNA-mediated MAVS inhibition could represent a possible approach for targeting MAVS. However, although siRNA-based strategies are being explored therapeutically, they still face significant challenges related to stability and efficient delivery. Therefore, while these results provide initial supportive evidence, further studies will be required to evaluate the feasibility of translating these findings into therapeutic applications.

## 5. Conclusions

In summary, for the first time, we demonstrated here that, in cancer, MAVS exerts broad effects by modulating protein networks that activate signaling pathways, ultimately leading to tumor suppression. This is very important, considering that mice lacking MAVS were viable, growing, and maturing normally [29], underscoring its cancer-specific role. MAVS depletion reduced proliferation, induced apoptosis, eliminated cancer stem-cells, reshaped the TME, and altered immune responses. Loss of MAVS enhanced CD-4^+^ infiltration, reduced PD-L1 expression, and increased tumor immunogenicity, suggesting that MAVS is a critical regulator of tumor-associated immunity and a potential target to improve immunotherapy strategies.

## Data Availability

All data generated and analyzed during the study are available in this paper in the main text and the Supplementary Materials.

## Supplementary Materials

The following supporting information can be downloaded at https://www.mdpi.com/article/10.3390/biom16040501/s1. Figure S1: Body weight of control and MAVS-KO PC-3 prostate cancer xenograft mice; Table S1: Antibodies used in this study; Table S2: Alterations in the expression of mitochondria-associated human proteins in MAVS-KO PC-3 human cancer cells; Table S3: Alterations in the expression of signaling-related human proteins in MAVS-KO PC-3 human cancer cells; Table S4: Alterations in the expression of immune-related human proteins in MAVS-KO PC-3 in human cancer cells; Table S5: Alterations in the expression of protein synthesis, degradation, and trafficking and their regulation-related proteins in MAVS-KO PC-3 human cancer cells; Table S6: Alterations in the expression of epigenetic and nuclear-related human proteins in MAVS-KO PC-3 human cancer cells; Table S7: Alterations in the expression of cytoskeletal and cell proliferation-related human proteins in MAVS-KO PC-3 human cancer cells; Table S8: Alterations in the expression of metabolism-related protein in MAVS-KO PC-3 human cancer cells. Original western blots can be found at Supplementary Materials.

## Abbreviations

The following abbreviations are used in this manuscript:

ATCC	American Type Culture Collection
CARDIF	Caspase activation recruitment domain adaptor inducing I FN-β
CARD	Caspase activation and recruitment domain
CSC	Cancer stem-cell
DAPI	4′,6-diamidino-2-phenylindole
ER	Endoplasmic reticulum
FBS	Fetal bovine serum
H&E	Hematoxylin-eosin
IHC	Immunohistochemistry
IF	Immunofluorescence
IFN	Interferon
IPS-1	IFN-β promoter stimulator I
IRF1	Interferon regulatory factor 1
IRF3	Interferon regulatory factor 3
MAVS	The mitochondrial anti-viral-signaling
MAM	Mitochondrial-associated membrane
Mfn2	Mitofusin 2
MS	Mass spectrometry
NF-κB	Nuclear factor **κ**B
NGS	Normal goat serum
OMM	Mitochondrial outer membrane
PBS	Phosphate-buffered saline
PD-L1	Programmed cell death ligand 1
RIG-I	Retinoic acid-inducible gene I
RLRs	RIG-I-like receptors
RPMI	Roswell Park Memorial Institute
TME	Tumor microenvironment
TUNEL	Terminal deoxynucleotidyl transferase dUTP nick end labeling
VISA	Virus-induced signaling adaptor
VDAC1	Voltage-dependent anion channel 1
YAP1	Yes-associated protein
