# Supplementary material for: MAVS as a Key Regulator of Tumor Proliferation, Survival, the Tumor Microenvironment, and Immunity
Source: Biomolecules. 2026 Mar 26;16(4):501. doi: 10.3390/biom16040501 (PMC13113892; doi:10.3390/biom16040501)

biomolecules

Supplementary Materials

Knockout of the mitochondrial anti-viral signaling protein MAVS in prostate cancer is associated with cell survival and tumor microenvironment alteration

Sweta Trishna[1], Anna Shteinfer-Kuzmine[2], Vered Chalifa-Caspi[3] and Varda Shoshan-Barmatz[1,2]#

[1]Department of Life Sciences and the [2]National Institute for Biotechnology in the Negev; [3]Ilse Katz Institute for Nanoscale Science and Technology, Ben-Gurion University of the Negev, Beer Sheva, 84105 Israel

biomolecules

MDPI

Table S1. Antibodies used in this study

Antibodies against the indicated protein, their catalogue number, source, and the dilutions used in IHC, immunoblot, and immunofluorescence experiments are presented.

Antibody	Source and Cat. No.		
		WB	IF
Mouse monoclonal anti-β-actin	Millipore, Billerica, MA, MAB1501	1:40000	
Rabbit polyclonal anti-MAVS	Abcam, Cambridge, UK, ab31334	1:2000	1:750
Mouse polyclonal anti-MAVS	Santa Cruz Biotechnology, TX, sc166583	-	1:500
Rabbit monoclonal anti-Ki-67	Abcam, Cambridge, UK, ab15580	-	1:500
Rabbit monoclonal anti-PD-L1	Abcam, Cambridge, UK, ab213524	1:1000	1:500
Rabbit polyclonal anti-INF-β	Abcam, Cambridge, UK, ab85803	-	1:500
Rabbit polyclonal anti-CD-31	Abcam, Cambridge, UK, ab28364	-	1:100
Rabbit polyclonal anti-CD44	Abcam, Cambridge, UK, ab157107	-	1:500
Rabbit monoclonal anti-CD4	Abcam, Cambridge, UK, ab183685	-	1:500
Mouse monoclonal anti-IFI-16	Santa Cruz Biotechnology, TX, USA, sc-8023	1:2000	1:500
Rabbit polyclonal anti-IFN-β	Abcam, Cambridge, UK, ab85803	-	1:500
Rabbit monoclonal anti-phospho-TBK1/NAK (Ser172)	Cell Signaling Tech, Davers, MA, 5483s	-	1:500
Rabbit monoclonal anti-YAP1	Abcam, Cambridge, UK, ab205270	-	1:500
Anti-rabbit IgG, HRP conjugate	Promega Corporation, Madison,USA, W4011	1:10000	-
Anti-mouse IgG, HRP conjugate	Abcam, Cambridge, UK, ab98799	1:10000	-
Anti-mouse IgG, Alexa Fluor 488	Abcam, Cambridge, UK, ab150109	-	1:800
Anti-mouse IgG, Alexa Fluor 555	Abcam, Cambridge, UK, ab 150110	-	1:1000
Anti-rabbit IgG, Alexa Fluor 555	Abcam, Cambridge, UK, ab150086	-	1:1000
Anti-rabbit IgG, Alexa Fluor 488	Thermo Fisher Scientific, USA, A-11008	-	1:1000

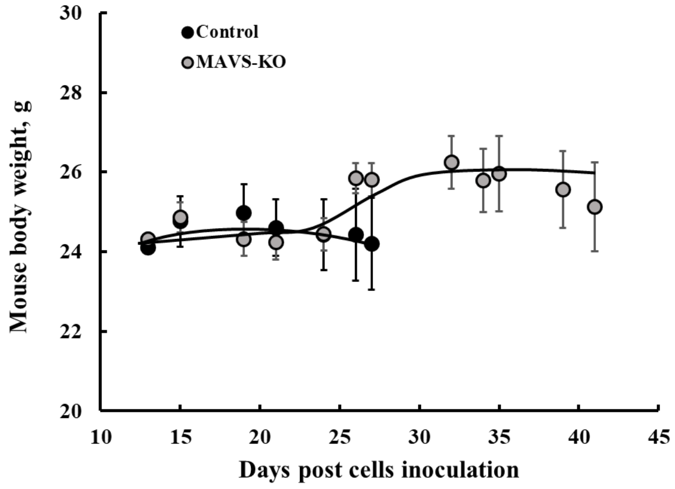

Figure S1. Body weight of control and MAVS-KO PC-3 prostate cancer xenograft mice. Control or MAVS-KO PC-3 cells (2×10^6 cells/mouse) were inoculated into athymic male mice. Mouse body weight was monitored during the course of the experiment, and the calculated averages are presented. Results represent the mean ± SEM (n = 5).

Table S2. Alterations in the expression of mitochondria-associated human proteins in MAVS-KO PC-3 human cancer cells

An LC-HR MS/MS analysis was performed as described in the Materials and Methods section. Differentially expressed proteins (*p*-value <0.01, |FC| > 1.5) in MAVS-KO cells vs. control are presented. For each protein, the name and linear fold change (FC) are provided, with plus and minus signs indicating up- and downregulation, respectively. The nominal *p*-value, as well as the protein's function, subcellular localization, and relevance to cancer are also indicated.

No.	Protein name (UniProtKB)	Proposed protein function (cellular localization)	FC (*p*-value)	Relation to disease
1.	**NADH-cytochrome b5 reductase 2 CYB5R2** Q6BCY4	NADH-cytochrome b5 reductase 2 (CYB5R2) is an enzyme involved in various cellular processes, including fatty acid desaturation, cholesterol biosynthesis, and drug metabolism[1, 2].	+30.05 (000139)	CYB5R2 has been identified as a potential tumor suppressor gene in human nasopharyngeal carcinoma (NPC) [2].
2.	**Sulfide:quinone oxidoreductase, mitochondrial SQOR** Q9Y6N5	SQOR catalyzes the oxidation of hydrogen sulfide with the help of a quinone, such as ubiquinone-10, giving rise to thiosulfate, and ultimately to sulfane (molecular sulfur) atoms [3]. (mitochondria)	+29.59 (0.002648)	Sulfide:quinone oxidoreductase-deficiency (SQORD) leads to episodes of encephalopathy and Leigh syndrome-like brain lesions, with acute symptoms triggered by infections and fasting [4].
3.	**ATP synthase F1 complex assembly facto 2 ATPAF2** Q8N5M1	ATPAF2 is a mitochondrial protein that plays a crucial role in the assembly of the F1 subunit of ATP synthase. (mitochondria)	+6.098 (002402)	Mutations in ATPAF2 have been associated with several mitochondrial disorders, notably mitochondrial Complex V deficiency, nuclear type 1 (MC5DN1) [5].
4.	**D-glutamate cyclase, mitochondrial DGLUCY** Q7Z3D6	D-glutamate cyclase is a mitochondrial enzyme encoded by the DGUC (also known as C10orf82) gene in humans.	+4.75 0.00788	Downregulation of this enzyme disrupts D-glutamate and 5-oxo-D-proline levels, which contribute to the onset or exacerbation of heart failure.
5.	**Carbonic anhydrase 5B, mitochondrial CA5B** Q9Y2D0	CA5B functions in reversible hydration of carbon dioxide for mitochondrial metabolic liver enzymes. (mitochondria)	-3.385 (0.00906)	Deficiency of bicarbonates, provided by CA5B for mitochondrial metabolic liver enzymes, leads to metabolic acidosis and hypoglycemia [6].
6.	**Mitochondrial antiviral-signaling protein MAVS** Q7Z434	MAVS is an adaptor protein in innate immune response against viral infection, cellular damage, and NLRP3-associated inflammation [7]. (mitochondria)	-5.951 (0.004186)	Overexpression of MAVS leads to autocrine inflammation in cancer, facilitating immune evasion by cancer cells.
7.	**Mitochondrial ribosome-. GTPase 2 MTG2** Q9H4K7	MTG2 plays a role in the regulation of the mitochondrial ribosome assembly and of translational activity [8].	-6.079 (0.000688)	Mutations in the MTG2 gene have been linked to COXPD19, a rare mitochondrial disorder.
8.	**39S ribosomal protein L42, mitochondrial MRPL42** Q9Y6G3	MRPL42 is a mitochondrial protein encoded by the nuclear gene and is responsible for protein synthesis in the mitochondria [9]. (mitochondria)	-22.061 (0.000081)	Knockdown of MRPL42 could reduce proliferation and colonization, and promote cell-cycle arrest in the G1/S phase, weakening the migration and invasion ability of cancer cells.

Table S3. Alterations in the expression of signaling-associated human proteins in MAVS-KO PC-3 human cancer cells

An LC-HR MS/MS analysis was performed as described in the Materials and Methods section. Differentially expressed proteins (*p*-value <0.01, |FC| > 1.5) in MAVS-KO cells vs. control are presented. For each protein, the name, fold change (FC), and *p*-value, as well as its function, subcellular localization, and relevance to cancer are indicated.

No.	Protein name (UniProtKB)	Proposed protein function (cellular localization)	FC (*p*-value)	Relation to disease
1.	**Insulin-like growth factor-binding protein 3 IGFBP3** P17936	IGF-binding proteins prolong the half-life of the IGFs, and inhibit or stimulate the growth promoting effects of the IGFs on cell culture [10]. (secreted)	+ 988.3 (0.000262)	IGFBP-3 is a highly effective pro-apoptotic factor in tumor cells, inducing therapy directly with exogenous IGFBP-3 or indirectly with hormonal or other up-regulators of IGFBP-3 [11].
2.	**[F-actin]-monooxygenase MICAL2** O94851	Methionine monooxygenase promotes depolymerization of F-actin by mediating oxidation of methionine residues 44 and 47 to form methionine-sulfoxide, resulting in actin filament disassembly and preventing repolymerization [12]. (cytoplasm, nucleus)	+90.02 (0.001238)	MICAL2 affects proliferation and cell migration, and controls muscle regeneration in cancer [13].
3.	**Glutamate receptor 2 GRIA2** P42262	L-glutamate acts as an excitatory neurotransmitter at many synapses in the central nervous system, and plays an important role in fast excitatory synaptic transmission [14]. (post-synaptic cell membrane)	+65.61 0.002644	Mutations in the GRIA2 gene, which encodes the glutamate ionotropic receptor AMPA Type Subunit 2 (GluA2), are linked to neurodevelopmental and neuropsychiatric disorders [15].
4.	**Plasminogen activator inhibitor 1 SERPINE1** P05121	SERPINE1 is a primary inhibitor of the tissue-type plasminogen activator (PLAT) and urokinase-type plasminogen activator (PLAU) [16]. (secreted)	+54.28 0.00401	Overexpression of PAI-1 leads to thrombophilia [17].
5.	**Rho GDP-dissociation inhibitor 2 ARHGDIB** P52566	RhoGDIβ regulates the GDP/GTP exchange reaction of the Rho proteins by inhibiting the dissociation of GDP from them, and the subsequent binding of GTP [18]. (cytosol)	+13.74 (0.000416)	RhoGDIβ is responsible for a reduction in lung cancer-cell invasion. It mediates ATG7-induced bladder cancer invasion [19].
6.	**C2 calcium-dependent domain-containing protein 4A C2CD4A** Q8NCU7	C2CD4A is involved in inflammatory processes and regulates cell architecture and adhesion [17]. (nucleus)	+10.37 (0.0008948)	C2CD4A is upregulated in response to inflammatory cytokines, such as IL-1β, in pancreatic islets and β-cells, indicating a role in the inflammatory processes associated with diabetes [20].
7.	**Serine/threonine-protein kinase 26 STK26** Q9P289	STK26 is a serine/threonine-protein kinase that acts as a mediator of cell growth and modulates apoptosis [21] (cytoplasm, Golgi apparatus)	+8.397 (0.00580)	STKs are part of the cell machinery involved in signal transduction pathways that control metabolism, cell division, angiogenesis, and other functions. They are hyper-activated in ovarian cancer [22, 23].
8.	**Integrin alpha-5 ITGA5** P08648	Integrin alpha-5 (ITGA5), also known as CD49e, is a transmembrane protein that plays a crucial role in cell adhesion, migration, and signaling [24]. (cell membrane, cell junction)	+ 7.48 (0.00794)	ITGA5 overexpression exerts a mitigating, manifested as increased viability, angiogenesis, mitochondrial function, and diminished apoptosis and oxidative stress [25].
9.	**Semaphorin-4B SEMA4B** Q9NPR2	SEMA4B inhibits axonal extension by providing local signals to specify territories inaccessible for growing axons [26]. (cell membrane)	+3.831 (0.007344)	SEMA4B expression was upregulated in lung adeno carcinoma (LUAD) tissues and mediated immune evasion of LUAD by increasing recruitment

				of immune-suppressive cells, such as regulatory T cells (Tregs) and myeloid-derived suppressor cells (MDSCs).
10.	**Retinoic acid-induced protein 3 GPRC5A** Q8NFJ5	GPRC5A is involved in modulating differentiation and maintaining the homeostasis of epithelial cells. It functions as a negative modulator of EGFR signaling, and may act as a lung tumor suppressor [27]. (cell membrane)	+3.649 (0.00467)	Retinoic acid-induced protein 3 overexpression can induce malignancy in hepatocellular carcinoma (HCC).
11.	**IQ domain-containing protein N IQCN** Q9H0B3	IQCN is essential for spermiogenesis and fertilization [28]. (mitochondrion, nucleus)	+3.416 (0.00463)	Homozygous variation of IQCN leads to abnormal acrosome structure and male infertility.
12.	**Adenylate cyclase type 10 ADCY10** Q96PN6	ADCY10 catalyzes the formation of the signaling molecule cAMP. It may function as a sensor that mediates responses to changes in cellular bicarbonate and CO2 levels, and it is involved in ciliary beat regulation [29]. (cytoplasm, cell membrane, cytoskeleton)	+3.109 (0.007)	It has a critical role in mammalian spermatogenesis by producing cAMP, which regulates cAMP-responsive nuclear factors indispensable for sperm maturation in the epididymis. It induces capacitation, the maturational process that sperm undergoes prior fertilization [30].
13.	**TOM1-like protein 1 TOM1L1** O75674	TOM1-like protein 1 is an adaptor protein involved in protein phosphorylation and signal transduction [31]. (Golgi apparatus)	-3.476 (0.003976)	TOM1 is associated with early-onset autoimmunity, antibody deficiency, and features of combined immunodeficiency [32].
14.	**Inositol-tetra-kisphosphate 1-kinase ITPK1** Q13572	ITPK1 is a multifunctional enzyme integral to inositol phosphate metabolism, influencing various cellular processes such as neural development, calcium signaling, apoptosis, and necroptosis. (cytoplasm)	-5.886 (0.002668)	Not reported
15.	**Protein S100-A6** P06703	Calcium sensor and modulator, contributing to cellular calcium signaling. (nucleus, cytosol, cell membrane)	-5.32 (0.005010)	Expressed in lung, colorectal, pancreatic, and liver cancers. Involved in nervous system diseases, leukemia, endometriosis, cardiovascular system diseases, osteoarthritis, and other related diseases [33].
16.	**Periplakin PPL** O60437	PPL acts as a localization signal in PKB/AKT-mediated signaling [34]. (cytoplasm)	-7.306 (0.005932)	High PPL expression is associated with poor prognosis in ovarian cancer patient [35].
17.	**RANBP2-type and C3HC4-type zinc finger containing 1 (XAP3, XAP4 HOIL1)** Q9BYM8	It enables several functions: transcription co-activator and ubiquitin binding activity, positive regulation of canonical NF-kappa B signal transduction, linear polyubiquitination, and regulation of DNA-binding transcription factor activity [36]. (cytoplasm)	-10.3 (0.0039)	RBCK1 mutations are a frequent cause of PGBM1, a glycogen storage disease characterized by the accumulation of polyglucosan bodies in tissues. This accumulation leads to progressive muscle weakness and cardiomyopathy [37].
18.	**Dedicator of cytokinesis protein 11 (DOCK11), also known as Zizimin2** Q5JSL3	DOCK11 is a member of the DOCK-D subfamily of the DOCK family of guanine nucleotide exchange factors (GEFs), which function as activators of small G-proteins. It activates the small G protein Cdc42 [38]. (cytoplasm)	-11.49 (00391)	Hemizygous DOCK11 mutations in humans are associated with early-onset and severe autoimmunity [39].
19.	**Sigma intracellular receptor 2 TMEM97** Q5BJF2	TMEM97 is an intracellular orphan receptor that binds various drugs. [40]. (nucleus membrane)	-21.48 (0.00033)	It is highly expressed in various proliferating cancer cells.

20.	**ProteinS100A2**<br>**S100-A2**<br>P29034	ProteinS100A2 functions as a calcium sensor and modulator in cellular calcium signaling. (nucleus, varies in different cell lines)	-69.19<br>(0.001808)	S100-A2 is overexpressed in multiple types of cancer and acts as a biomarker of poor prognosis in colorectal cancer [41].

Table S4. Alterations in the expression of immune system-associated human proteins in MAVS-KO PC-3 in human cancer cells
An LC-HR MS/MS analysis was performed as described in the Materials and Methods section. Differentially expressed proteins (p-value <0.01, |FC| > 1.5) in MAVS-KO cells vs. control are presented. For each protein, the name, fold change (FC), and p-value, as well as its function, subcellular localization, and relevance to cancer are indicated.

No.	Protein name (UniProtKB)	Proposed protein function (cellular localization)	FC (p-value)	Relation to disease
1.	**Interleukin-1 beta**<br>**IL1B**<br>P01584	IL-1β is a key pro-inflammatory cytokine involved in innate immune response. (cytoplasm)	+48.77<br>(0.003722)	A positive feedback loop exists between IL-1β and IL-6, which helps form a continuous local inflammatory, microenvironment, and further amplifies the inflammatory response in the intervertebral disc [42].
2.	**Gamma-interferon-inducible protein 16**<br>**IFI16**<br>Q16666	IFI16 binds double-stranded supercoiled DNA and cruciform DNA structures, and is involved in transcriptional regulation, and in innate immune response by recognizing viral dsDNA in the cytosol and in the nucleus. Binding viral DNA results in recruitment of TMEM173/ STING, thereby leading to the induction of IFN-beta and other interferon-related cytokines [43]. (cytoplasm, nucleus)	+32.58<br>(0.0007214)	Abnormal IFI16 expression is closely associated with immune system diseases and the occurrence of various malignant tumors such as systemic lupus erythematosus and breast cancer. Increased expression of IFI16 protein in LNCaP prostate cancer cells downregulates AR expression and inhibits AR-mediated functions [44].
3.	**Plasminogen activator inhibitor 2**<br>**SERPINB2**<br>P05120	Serine protease inhibitor, involved in inflammation, immune responses, and regulation of plasminogen activation. Inhibits urokinase-type plasminogen activator. (cytoplasm)	+13.95<br>(0.000657)	PAI-2 is elevated during in pneumococcal meningitis in humans and mice. PAI-2 deficiency causes an inflammatory imbalance, resulting in increased brain pathology and mortality [45]
4.	**CKLF-like MARVEL transmembrane domain**<br>**CMTM6**<br>Q9NX76	CMTM6 is a master regulator of expression and recycling of PD-L1/CD274, an immune inhibitory ligand critical for immune tolerance to self and antitumor immunity [46]. (cytoplasm and cell membrane)	+9.033<br>(0.000968)	It associates with both constitutive and IFNG-induced PD-L1/CD274 at recycling endosomes, where it protects PD-L1/CD274 from being targeted for lysosomal degradation [47].
5.	**Dipeptidyl peptidase 4**<br>**DPP4**<br>P27487	DPP4 is a cell surface glycoprotein receptor involved in the co-stimulatory signal essential for T-cell receptor- mediated T-cell activation [48]. It is involved in the promotion of lymphatic endothelial cells, and adhesion and migration [49]. (cell membrane)	-3.623<br>(0.00215)	DPP4 is a cell surface protein that can act as a tumor suppressor or activator, depending upon the level of expression and interaction with the microenvironment and chemokines [50].
6.	**TRAF-type zinc finger domain-containing protein 1**	TRAFD1 is a negative feedback regulator that controls excessive innate immune responses. It	-3.912<br>(0.008678)	TRAFD1 interacts with miR-34a and acts as a major hub of T-cell regulatory networks, suggesting that

	**TRAFD1** O14545	regulates both Toll-like receptor 4 (TLR4) and DDX58/RIG1-like helicase (RLH) pathways. It negatively regulates the RLH pathway downstream from MAVS and upstream of NF-kappa-B and IRF3 [51]. (mitochondrion)		it utilizes miR-34a as a target of intervention towards immune responsiveness modulation of T cells [52].
7.	**Myeloid differentiation primary response protein MYD88** Q99836	is an adapter protein involved in the Toll-like receptor and IL-1 receptor signaling pathways in the innate immune response [53]. (cytoplasm, nucleus)	-5.562 (0.002168)	MYD88 induces NLRP3- and NF-κB- associated inflammation upon viral infection like SAR-COV2, SAR-COV1, and HIV.
8.	**Ubiquitin thioesterase otulin OTULIN** Q96BN8	OTULIN deubiquitinase specifically removes linear ('Met-1'-linked) polyubiquitin chains and acts as a regulator of angiogenesis and the innate immune response [54]. (cytoplasm)	-6.455 (0.002102)	Mutation of OTULIN causes OTULIN-related autoinflammatory syndrome (ORAS) in humans [55].
9.	**Lymphoid-specific helicase HELLS** Q9NRZ9	HELLS is involved in regulating the survival of lymphoid cells. It is required for maintenance DNA methylation. (nucleus)	-6.832 (0.003296)	HELLS promotes cervical cancer proliferation by inhibiting nuclear factor erythroid 2-related factor 2 (Nrf2) expression [56].

Table S5. Alterations in the expression of protein synthesis, degradation, trafficking and their regulation-associated human proteins in MAVS-KO PC-3 human cancer cells
An LC-HR MS/MS analysis was performed as described in the Materials and Methods section. Differentially expressed proteins (p-value <0.01, |FC| > 1.5) in MAVS-KO cells vs. control are presented. For each protein, the name, fold change (FC), and p-value, as well as its function, subcellular localization, and relevance to cancer are indicated.

No.	Protein name (UniProtKB)	Proposed protein function (cellular localization)	FC (p-value)	Relation to disease
1.	**T-complex protein 1 subunit zeta-2 CCT6B** Q92526	CCT6B is a component of the chaperonin-containing T-complex (TRiC), a molecular chaperone complex that assists in the folding of proteins [57]. (cytoplasm)	+83.46 (0.000037)	CCT6A levels significantly increase in the plasma of systemic lupus erythematosus (SLE) and rheumatoid arthritis patients [58].
2.	**Stonin-2 STON2** Q8WXE9	STON2 is an adapter protein involved in endocytic machinery and synaptic vesicle recycling. It may facilitate clathrin-coated vesicle uncoating [59]. (cytoplasm)	+14.66 (0.0001156)	Elevated STON2 expression in epithelial ovarian cancer correlates with unfavorable clinical features, including intraperitoneal metastasis, platinum resistance, and recurrence [59].
3.	**B-cell receptor-associated protein 29 BCAP29** Q9UHQ4	BCAP29 comprises integral membrane proteins with a role in ER quality control and sorting [60]. (cytoplasm)	+7.283 (0.0042)	Because it is required by both tumor and normal cells like housekeeping, BCAP29 cannot be a biomarker for cancer [61].
4.	**YIF1B protein** Q5BJH7	YIF1B is a membrane trafficking protein crucial for vesicle-mediated transport between the endoplasmic reticulum and the Golgi apparatus. (ER, golgi)	+5.867 0.002446	YIF1B is involved in serotonin receptor (HTR) membrane trafficking and signal transmission in neuropathy [62].
5.	**Ras-related protein Rab-3B RAB3B** P20337	RAB3B is a member of the RAB family of small GTPases, which are key regulators of vesicle trafficking in cells. (cytoskeleton)	+4.746 0.003174	In HCC, RAB3B is upregulated in cancer stem-like cells and is associated with chemoresistance and metastatic potential [63].
6.	**Intersectin-2 ITSN2** Q9NZM3	Intersectin-2 is primarily categorized as a scaffold or adaptor protein [64]. (cytoplasm)	+3.089 (0.006328)	Deletion of ITSN1/2 in mice leads to stereotypic behavior, morphological abnormalities, and defective cortico-striatal neurotransmission linked to NMDA receptor currents [65].

7.	**Heat shock-related protein 2 HSPA2** P54652	HSPA2 assists in the proper folding of nascent proteins and the refolding or degradation of misfolded proteins, maintaining cellular proteostasis. (cytoplasm)	+2.611 (0.004962)	HSPA2 is upregulated in pancreatic cancer, and is closely associated with tumor immunity and aggressive progression [66].
8.	**Golgin subfamily A member 7 GOLGA7** Q7Z5G4	GOLGA7 plays a crucial role in transporting proteins from the Golgi apparatus to the plasma membrane [67]. (Golgi apparatus)	-3.707 (0.008124)	A polymorphism (rs11337) in the GOLGA7 gene, located at a microRNA binding site, has been associated with glioma prognosis [68].
9.	**Ubiquitin-associated domain-containing protein 1 UBAC1** Q9BSL1	UBAC1 is a non-catalytic component of the KPC complex, a E3 ubiquitin-protein ligase complex that mediates polyubiquitination of target proteins such as CDKN1B and NFKB1 [69, 70]. (cytoplasm)	−3.456 (0.011072)	High UBAC1 expression is associated with a favorable prognosis in cervical cancer [71].
10.	**Endoplasmic reticulum transmembrane helix translocase ATP13A1** Q9HD20	ATP13A1 is required to remove mitochondrial transmembrane proteins mistargeted to the endoplasmic reticulum. (ER)	-2.391 (0.00657)	ATP13A1 is essential for MAVS stability and activation. Its loss leads to the degradation of MAVS, impairing the production of type I interferons and compromising cellular anti-viral defense mechanisms. Cell deficient in ATP13A1 exhibits susceptibility to RNA virus infections [72].
11.	**Reticulocalbin-3 RCN3** Q96D15	RCN3 is molecular chaperone-assisting protein biosynthesis and transport in the endoplasmic reticulum [73]. (ER)	-3.588 (0.00701)	In-silico analysis of glioblastoma patient datasets demonstrate a positive correlation of RCN3 with ribosomal pathway genes [74].
12.	**Trafficking protein particle complex subunit 5 TRAPPC5** Q8IUR0	TRAPPC5 plays a role in vesicular transport from the endoplasmic reticulum to the Golgi [75]. (ER, Golgi apparatus)	-3.999 (0.007374)	No direct impact on any disease has been reported.
13.	**Torsin-1A TOR1A** O14656	TOR1A is primarily localized in the ER and the peri-nuclear space, where it plays a critical role in maintaining nuclear envelope integrity, facilitating protein trafficking, and regulating synaptic function [76]. (ER, nuclear membrane)	-7.308 (0.001356)	Not reported

Table S6. Alterations in the expression of epigenetic and nuclear-associated human proteins in MAVS-KO PC-3 human cancer cells

An LC-HR MS/MS analysis was performed as described in the Materials and Methods section. Differentially expressed proteins (p-value <0.01, |FC| > 1.5) in MAVS-KO cells vs. control are presented. For each protein, the name, fold change (FC), and p-value, as well as its function, subcellular localization, and relevance to cancer are indicated.

No.	Protein name (UniProtKB)	Proposed protein function (cellular localization)	FC (p-value)	Relation to disease
1.	**Myelin expression factor 2 MYEF2** Q9P2K5	MYEF2 is a transcriptional repressor that mainly inhibits the transcription of the myelin basic protein gene (MBP) by binding its promoter, thereby participating in brain development [77]. (nucleus)	+5.279 (0.00235)	High MYEF2 expression is an independent prognostic factor for patients with hepatocellular carcinoma (HCC). Elevated MYEF2 facilitated cell migration and invasion in vitro [78].
2.	**Terminal uridylyltransferase 7 TUT7** Q5VYS8	TUT7 plays a pivotal role in RNA metabolism by adding uridine residues to the 3' ends of RNA molecules in a process known as uridylation. (cytoplasm)	+4.452 (0.00111)	TUT7 functions as a regulator in TLR4-driven inflammatory responses by mediating uridylation, thus, destabilizing the mRNAs of inflammatory mediators [79].
3.	**Protein SCAI SCAI**	SCAI is a tumor suppressor that suppresses MRTFA-induced SRF	+4.459 (0.00497)	High expression of SCAI correlated with better survival in patients with

	Q8N9R8	transcriptional activity. It functions in the RHOA-DIAPH1 signal transduction pathway and regulates cell migration via transcriptional regulation of ITGB1 [80]. (nucleus, cytoplasm)		breast and lung cancers, in contrast to other cancers (gastric, prostate, colorectal), where high SCAI expression correlated with poor survival of patients [81].
4.	**Zinc finger CCCH domain-containing protein 7A** **ZC3H7A** Q8IWR0	This is a specific regulator of miRNA biogenesis that binds to microRNAs MIR7-1, MIR16-2, and MIR29A hairpins [82]. (nucleus)	+4.299 (0.00595)	Translocation of the ZC3H7A gene is present in non-small cell lung carcinoma patients and can act as a prognostic marker [83].
5.	**Surfeit locus protein 2** **SURF2** Q15527	SURF2 is involved in RNA processing, ribosome biogenesis, and nuclear structure. (nucleus, plasma membrane)	+4.215 (0.005798)	Not reported
6.	**Ashwin** **C2orf49** Q9BVC5	C2orf49 is a component of the tRNA-splicing ligase complex, which plays a crucial role in the ligation step of tRNA splicing [84]. (nuleoplasm)	+3.555 (0.005748)	No direct impact has been reported.
7.	**Core histone macro-H2A.2** **MACROH2A2** Q9P0M6	This variant of histone H2A replaces conventional H2A in a subset of nucleosomes, where it represses transcription [85]. (Nucleus)	+2.989 (0.002882)	The dysregulated expression of macro H2A histone variants has been observed as a poor prognostic marker for hepatocellular carcinoma, breast cancer, colon cancer, melanoma [86].
8.	**Protein C-ets-1** **ETS1** P14921	ETS1 is a transcription factor that directly controls the expression of cytokines and chemokine genes in a wide variety of different cellular contexts [87]. (cytoplasm)	+2.916 (0.004072)	ETS1 regulates angiogenesis through regulation of the expression of genes controlling endothelial cell migration and invasion in cancer [88].
9.	**Disco-interacting protein 2 homolog B** **DIP2B** Q9P265	DIP2B is a gene involved in various biological processes that include DNA methylation, cell proliferation, and cancer progression. (cytoplasm)	+2.26 0.00953	DIP2B plays multiple roles in cell proliferation, migration, and apoptosis during embryogenesis, and may control metabolism in lung fibroblasts [89].
10.	**Histone H1.10** **H1–10** Q92522	Histones of H1 are necessary for the condensation of nucleosome chains into higher-order structures [90]. (nucleus)	-2.539 (0.00678)	These are highly expressed in neuroendocrine tumors [91].
11.	**Transmembrane protein 199** **TMEM199** Q8N511	Nuclear TMEM199 regulates CD274 mRNA expression. (cytoplasm, ER)	-2.693 0.009536	Nuclear-located TMEM199 regulates PD-L1 mRNA levels by binding to transcription factors such as IFNGR1, IRF1, MTMR9, and Trim28, which all promote PD-L1 mRNA expression [92].
12.	**Transmembrane protein 179B** **TMEM179B** Q7Z7N9	TMEM179B is proposed to be functional in kidney renal cell carcinoma (Human Atlas). (nuclear speckels)	-3.003 (0.008216)	It is proposed to be functional in kidney renal cell carcinoma.
13.	**TruB pseudouridylate synthase** **TRUB1** Q8WWH5	TRUB1, also known as PUS4, is a highly conserved enzyme in humans that plays a pivotal role in RNA modification, particularly in pseudouridylation [93]. (cytoplasm, nucleus)	-4.309 (0.005534)	TruB1 acts suppressively on cell proliferation due to the selective promotion of let-7 maturation, whereas PUS10 tends to promote cell proliferation [94].
14.	**Zinc finger protein 706** **ZNF706** Q9Y5V0	Cys2-His2 zinc-finger proteins (C2H2-ZNFs) constitute the largest class of DNA-binding transcription factors interacting with and regulating RNA-associated processes [92]. (cytoplasm, nucleus)	-7.946 (0.0007318)	ZNF703 regulates luminal B cancer stem cells (CSCs) via transcriptional control of key cellular processes. High expression of ZNF706 is associated with poor survival in HCC patients [95].

Table S7. Alterations in the expression of cell proliferation and cytoskeletal-associated human proteins in MAVS-KO PC-3 human cancer cells

An LC-HR MS/MS analysis was performed as described in the Materials and Methods section. Differentially expressed proteins (*p*-value <0.01, |FC| > 1.5) in MAVS-KO cells vs. control are presented. For each protein, the name, fold change (FC), and *p*-value, as well as its function, subcellular localization, and relevance to cancer are indicated.

No.	Protein name (UniProtKB)	Proposed protein function (cellular localization)	FC (*p*-value)	Relation to disease
1.	**Tubulin alpha-1A chain TUBA1A** Q71U36	TUBA1A encodes the alpha-1A isoform of tubulin, a component of microtubules, maintaining cell shape, enabling intracellular transport, and segregating chromosomes during cell division, cell adhesion, and cell movement [96]. It is essential for neuronal migration during brain development. (cytoskeleton)	+8.312 (0.00279)	TUBA1A mutations account for approximately 5% of lissencephaly (LIS) cases [97].
2.	**Synaptopodin SYNPO** Q8N3V7	SYNPO is an actin-associated protein encoded by the SYNPO gene. It plays a crucial role in modulating the actin cytoskeleton in both neuronal dendritic spines and the kidneys. (cytoplasm)	+5.532 (0.00333)	In the brain, synaptopodin is predominantly expressed in telencephalic neurons, including regions such as the hippocampus, cerebral cortex, striatum, and olfactory bulb [98].
3.	**Dynein light chain Tctex-type 1 DYNLT1** P63172	DYNLT1 is a protein-coding gene that encodes a component of the cytoplasmic dynein motor complex. (cytoskeleton)	+4.637 (0.000842)	DYNLT1 is upregulated in breast tumors, particularly, in estrogen receptor-positive and triple-negative subtypes. It promotes proliferation, migration, invasion, and mitochondrial metabolism in breast cancer cells [99].
4.	**Brain acid soluble protein 1 BASP1** P80723	BASP1 acts as a transcriptional co-suppressor for the Wilms' tumor suppressor protein WT1. (cell membrane)	+4.593 (0.008364)	BASP1 knockdown inhibited the proliferation of cervical cancer, suppressed cell-cycle progression, and decreased tumorgenicity [100].
5.	**Filamin-C FLNC** Q14315	Filamin C (FLNC) is a muscle-specific protein encoded by the FLNC gene, that plays a crucial role in the structural integrity and function of striated muscles, particularly, in the heart and skeletal muscles. (cytoplasm)	+4.258 (0.00145)	Missense mutations in FLNC, particularly within the ROD2 domain, are associated with hypertrophic cardiomyopathy (HCM) [101].
6.	**Myosin-8 MYH8** P13535	MYH8 (myosin heavy chain 8) is a gene encoding a class II myosin heavy chain protein, predominantly expressed during fetal skeletal muscle development. (cytoplasm)	+4.118 (0.009172)	Not reported
7.	**Laminin subunit beta-3 LAMB3** Q13751	LAMB3 facilitates cell adhesion, migration, and differentiation. (extracellular matrix)	+3.745 (0.00942)	LAMB3 mediates apoptotic, proliferative, invasive, and metastatic behaviors in pancreatic cancer by regulating the PI3K/ Akt signaling pathway [102].
8.	**Leucine-rich repeat and calponin and homology domain-containing protein 2 LRCH2** Q5VUJ6	LRCH2 plays a role in the organization of the cytoskeleton. (cytoplasm)	+3.404 (0.007652)	Mutations in leucine-rich repeat kinase 2 (LRRK2) are the most common genetic cause of Parkinson's disease [103].

9.	**Adenylyl cyclase-associated protein 2** **CAP2** P40123	CAP2 is involved in the regulation of actin polymerization. (cell membrane)	+2.841 (0.004212)	In CAP2-deficient mice, keratinocytes show reduced migration velocity and delayed wound closure [104].
10.	**Actin filament-associated protein 1** **AFAP1** Q8N556	AFAP1 is an adaptor protein that links signaling molecules to the actin cytoskeleton. (cytoplasm)	+2.696 (0.005084)	High expression of AFAP1 antisense RNA 1 was associated with poor prognosis in digestive system cancers [105].
11.	**Leupaxin** **LPXN** O60711	LPXN is a transcriptional coactivator for androgen receptor (AR) and serum response factor (SRF). It contributes to the regulation of cell adhesion, spreading and cell migration, and acts as a negative regulator in integrin-mediated cell adhesion events [106]. (cytoplasm, nucleus, cell membrane)	+2.575 (0.00576)	LPXN dysregulation was shown to participate in tissue invasion by promoting cancerous cell adhesion and migration [107].
12.	**Podocalyxin** **PODXL** O00592	PODXL is involved in the regulation of both adhesion and cell morphology, and in cancer progression. (cell membrane)	+2.598 (0.005544)	Human PODXL enhances the adherence of cells to immobilized ligands and to vascular endothelial cells through a mechanism(s) dependent on the activity of integrins [108].
13.	**Junctional adhesion molecule A** **F11R** Q9Y624	F11R, also known as junctional adhesion molecule-A (JAM-A) or CD321, is a transmembrane protein belonging to the immunoglobulin superfamily. It plays a pivotal role in maintaining epithelial and endothelial cell junction integrity, regulating leukocyte transmigration, and modulating platelet function. (cell membrane)	+2.531 (p=0.010000)	F11R silencing led to decreased cell proliferation, a loss of cell invasiveness, cell-cycle arrest in the G1 phase, and enhanced cell apoptosis in pancreatic cancer [109].
14.	**Tensin-3** **TNS3** Q68CZ2	Act as a protein phosphatase and/or a lipid phosphatase. Involved in the dissociation of the integrin-tensin-actin complex and in regulating cell migration and proliferation [110].	+2.34 0.008302	Tensin 3 suggested to act as a tumour suppressor [111].
15.	**Ubiquitin-conjugating enzyme E2 R2** **UBE2R2** Q712K3	This is a protein phosphatase inhibitor that specifically inhibits phosphatase 2A (PP2A) during mitosis. UBE2R2 is essential for keeping cyclin-B1-CDK1 activity high during the M phase [112]. (cytoplasm)	-2.324 (0.009502)	Diseases associated with UBE2R2 include clear cell adenofibroma [113].
16.	**Mitotic interactor and substrate of PLK1** **MISP** Q8IVT2	MISP (mitotic spindle positioning), also known as C19orf21, is an actin-binding protein integral to mitotic spindle orientation, cell division, and cytoskeletal organization. (cytoplasm)	-2.803 0.009322	MISP was found to promote cell proliferation in vitro and tumorigenesis in vivo [114].
17.	**Cordon-bleu protein-like 1** **COBLL1** Q53SF7	COBLL1 is a protein involved in actin cytoskeleton remodeling and pathological processes, including cancer progression, metabolic disorders, and developmental anomalies (cytoplasm)	-3.812 (0.003722)	High COBLL1 expression correlates with poor prognosis in chronic lymphocytic leukemia (CLL) patients [115].

Table S8. Alterations in the expression of metabolism-associated human proteins in MAVS-KO PC-3 human cancer cells

An LC-HR MS/MS analysis was performed as described in the Materials and Methods section. Differentially expressed proteins (*p*-value <0.01, |FC| > 1.5) in MAVS-KO cells vs. control are presented. For each protein, the name, fold change (FC), and *p*-value, as well as its function, subcellular localization, and relevance to cancer are indicated.

No.	Protein name (UniProtKB)	Proposed protein function (cellular localization)	FC (*p*-value)	Relation to disease
1.	**Creatine kinase M-type CKM** P06732	CKM plays a vital role in cellular energy homeostasis, such as in skeletal and cardiac muscles [116]. (cytoplasm)	+ 50.35 (0.0005626)	CKM is used to diagnose muscular dystrophy [117].
2.	**Perilipin-2 PLIN2**	Perilipins (PLINs) coat the surface of lipid droplets and are important for the regulation of lipid turnover [118]. (cell membrane, cytoplasm)	+20.78 (0.000712)	Plin2 liver-specific ablation alleviates diet-induced hepatic steatosis and inflammation.[118]
3.	**Dehydrogenase/reductase SDR family member 9 DHRS9** Q9BPW9	DHRS9 belongs to the short-chain dehydrogenase/reductase family. It plays a pivotal role in retinoid metabolism and steroid hormone processing. (ER)	+15.33 (0.008292)	DHRS9 expression may serve as a predictive and prognostic biomarker for rectal cancer patients who have undergone neo-adjuvant concurrent chemoradiotherapy (CCRT) [119].
4.	**Aldo-keto reductase family 1 member C3 AKR1C3** P42330	Aldo-keto reductase family 1 member C3 (AKR1C3) is an enzyme encoded by the AKR1C3 gene in humans [120]. (cytoplasm)	+ 3.998 (0.006266)	AKR1C3 a biomarker for active intratumoral steroidogenesis, and it has been measured in biopsies and transurethral resections of prostate specimens [120].
5.	**Aldo-keto reductase family 1 member C1 AKR1C1** Q04828	This enzyme plays a significant role in steroid hormone metabolism. (cytoplasm)	+3.603 (0.004114)	AKR1C1 is upregulated in metastatic bladder cancer cells, enhancing their invasive potential [121].
6.	**Alpha-(1,6)-fucosyltransferase FUT8** Q9BYC5	FUT8 is involved in the biosynthesis of glycoproteins such as glycosyltransferase. (Golgi complex)	+3.302 (0.00353)	Aberrant expression of FUT8 coincides with an increase in tumor metastasis [122].
7.	**Serpin B8 SERPINB8** P50452	SERPINB8 is a member of the serpin (serine protease inhibitor) superfamily which acts as an irreversible inhibitor of serine proteases. (cytoplasm)	+2.941 (0.003102)	The A329S variant of serpinB9 exhibits unobstructed granzyme B inhibition, but compromised caspase-1 inhibition [123].
8.	**Serine racemase SRR** Q9GZT4	SRR catalyzes the synthesis of D-serine from L-serine [124]. (cytoplasm)	+2.228 (0.008864)	In colorectal cancer, its expression is upregulated compared to normal mucosa [89].
9.	**Carboxy-methylenebutenolidase homolog CMBL** Q96DG6	CMBL is a cysteine hydrolase of the dienelactone hydrolase family that is highly expressed in liver cytosol. CMBL cleaves cyclic esters [125]. (cytoplasm)	-2.508 (0.005532)	Carboxymethyl-enebutenolidase acts as a tumor suppressor by re-programming glycolysis in colorectal cancer (CRC) [126].
10.	**Guanylate kinase GUK1** Q16774	GUK1 catalyzes the phosphorylation of GMP to GDP. It is essential for recycling GMP and, indirectly, cGMP [127]. (cytoplasm)	-2.558 (0.007552)	GUK1 deficiency is a potentially treatable cause of mitochondrial DNA (mtDNA) depletion/deletion syndrome (MDDS) [128].
11.	**CMP-N-acety ineuraminate-beta-galactosamide-alpha-2,3-sialyltransferase 4 ST3GAL4** Q11206	It is a beta-galactoside alpha2-3 sialyltransferase involved in terminal sialylation of glycoproteins and glycolipids [129]. (Golgi apparatus)	-2.621 (0.010416)	In breast cancer, it contributes to tumor progression by modifying cell surface glycoproteins, thereby altering cell signaling and cell–matrix interactions [130].

12.	**Glutamine synthetase** **GLUL** P15104	GLUL facilitates the production of glutamine, essential for nucleotide biosynthesis, energy production, and redox balance in rapidly proliferating cells. (cytoplasm, cell membrane)	-3.291 (0.002142)	Elevated GLUL expression in breast cancer correlates with larger tumor size and higher HER2 levels [131].
13.	**Putative monooxygenase p33MONOX** **KIAA1191** Q96A73	It is a potential NADPH-dependent oxidoreductase that is involved in the regulation of neuronal survival, differentiation, and axonal outgrowth. (cytoplasm)	-4.799 (0.00439)	KIAA1191's high expression suppressed the proliferation and migration of multiple myeloma (MM) cells [132].
14.	**Cystathionine beta-synthase** **CBS** P35520	CBS, with a pivotal position in mammalian sulfur metabolism at the homocysteine, acts through control of Hcy, H_2S, and GSH metabolism, and exerts diverse biological functions including regulating DNA methylation, mitochondrial respiration, and redox homeostasis [133]. (cytoplasm, nucleus)	-10.54 (0.000724)	Mutations in CBS are the single most common cause of hereditary hyperhomocysteinemia, with cardiovascular complications, but it also affects the ocular, central nervous, and skeletal systems.
15.	**Perilipin-4** **PLIN4** Q96Q06	PLINs coat the surface of lipid droplets and are important for the regulation of lipid turnover [118]. (cell membrane, cytoplasm)	-13.44 (0.00295)	PLIN4 is highly expressed in luminal A and B breast carcinomas, and its expression is associated with triple-negative breast cancer resistance to cytotoxic chemotherapy [134].

References

1. Arnoult, D., F. Soares, I. Tattoli, C. Castanier, D.J. Philpott, and S.E. Girardin, *An N-terminal addressing sequence targets NLRX1 to the mitochondrial matrix.* J Cell Sci, 2009. **122**(Pt 17): p. 3161-8.
2. Xiao, X., W. Zhao, F. Tian, X. Zhou, J. Zhang, T. Huang, B. Hou, C. Du, S. Wang, Y. Mo, et al., *Cytochrome b5 reductase 2 is a novel candidate tumor suppressor gene frequently inactivated by promoter hypermethylation in human nasopharyngeal carcinoma.* Tumour Biol, 2014. **35**(4): p. 3755-63.
3. Jackson, M.R., S.L. Melideo, and M.S. Jorns, *Human sulfide:quinone oxidoreductase catalyzes the first step in hydrogen sulfide metabolism and produces a sulfane sulfur metabolite.* Biochemistry, 2012. **51**(34): p. 6804-15.
4. Friederich, M.W., A.F. Elias, A. Kuster, L. Laugwitz, A.A. Larson, A.P. Landry, L. Ellwood-Digel, D.M. Mirsky, D. Dimmock, J. Haven, et al., *Pathogenic variants in SQOR encoding sulfide:quinone oxidoreductase are a potentially treatable cause of Leigh disease.* J Inherit Metab Dis, 2020. **43**(5): p. 1024-1036.
5. Zhou, Z., K. Zhang, Z. Liu, X. Gao, K. Huang, C. Chen, D. Wang, Q. Yang, and Q. Long, *ATPAF1 deficiency impairs ATP synthase assembly and mitochondrial respiration.* Mitochondrion, 2021. **60**: p. 129-141.
6. Diez-Fernandez, C., V. Rüfenacht, S. Santra, A.M. Lund, R. Santer, M. Lindner, T. Tangeraas, C. Unsinn, P. de Lonlay, A. Burlina, et al., *Defective hepatic bicarbonate production due to carbonic anhydrase VA deficiency leads to early-onset life-threatening metabolic crisis.* Genetics in Medicine, 2016. **18**(10): p. 991-1000.
7. Trishna, S., A. Lavon, A. Shteinfer-Kuzmine, A. Dafa-Berger, and V. Shoshan-Barmatz, *Overexpression of the mitochondrial anti-viral signaling protein, MAVS, in cancers is associated with cell survival and inflammation.* Molecular Therapy - Nucleic Acids, 2023. **33**: p. 713-732.
8. Hirano, Y., R.L. Ohniwa, C. Wada, S.H. Yoshimura, and K. Takeyasu, *Human small G proteins, ObgH1, and ObgH2, participate in the maintenance of mitochondria and nucleolar architectures.* Genes to Cells, 2006. **11**(11): p. 1295-1304.
9. Jiang, W., C. Zhang, Y. Kang, X. Yu, P. Pang, G. Li, and Y. Feng, *MRPL42 is activated by YY1 to promote lung adenocarcinoma progression.* J Cancer, 2021. **12**(8): p. 2403-2411.
10. Muzumdar, R.H., D.M. Huffman, G. Atzmon, C. Buettner, L.J. Cobb, S. Fishman, T. Budagov, L. Cui, F.H. Einstein, and A. Poduval, *Humanin: a novel central regulator of peripheral insulin action.* PloS one, 2009. **4**(7): p. e6334.
11. Jogie-Brahim, S., D. Feldman, and Y. Oh, *Unraveling Insulin-Like Growth Factor Binding Protein-3 Actions in Human Disease.* Endocrine Reviews, 2009. **30**(5): p. 417-437.
12. Lundquist, M.R., A.J. Storaska, T.C. Liu, S.D. Larsen, T. Evans, R.R. Neubig, and S.R. Jaffrey, *Redox modification of nuclear actin by MICAL-2 regulates SRF signaling.* Cell, 2014. **156**(3): p. 563-76.
13. Giarratana, N., F. Conti, R. La Rovere, R. Gijsbers, P. Carai, R. Duelen, T. Vervliet, G. Bultynck, F. Ronzoni, R. Piciotti, et al., *MICAL2 is essential for myogenic lineage commitment.* Cell Death & Disease, 2020. **11**(8): p. 654.
14. Kolleker, A., J.J. Zhu, B.J. Schupp, Y. Qin, V. Mack, T. Borchardt, G. Köhr, R. Malinow, P.H. Seeburg, and P. Osten, *Glutamatergic Plasticity by Synaptic Delivery of GluR-B(long)-Containing AMPA Receptors.* Neuron, 2003. **40**(6): p. 1199-1212.

15. Salpietro, V., C.L. Dixon, H. Guo, O.D. Bello, J. Vandrovcova, S. Efthymiou, R. Maroofian, G. Heimer, L. Burglen, S. Valence, et al., *AMPA receptor GluA2 subunit defects are a cause of neurodevelopmental disorders.* Nature Communications, 2019. **10**(1): p. 3094.
16. Jankun, J., A.M. Aleem, S.H. Selman, E. Skrzypczak-Jankun, W. Lysiak-Szydlowska, N. Grafos, H.J.L. Fryer, and R.S. Greenfield, *Highly stable plasminogen activator inhibitor type one (VLHL PAI-1) protects fibrin clots from tissue plasminogen activator-mediated fibrinolysis.* International journal of molecular medicine, 2007. **20**(5): p. 683-687.
17. Morange, P.E., N. Saut, M.C. Alessi, J.S. Yudkin, M. Margaglione, G. Di Minno, A. Hamsten, S.E. Humphries, D.A. Tregouet, and I. Juhan-Vague, *Association of Plasminogen Activator Inhibitor (PAI)-1 (SERPINE1) SNPs With Myocardial Infarction, Plasma PAI-1, and Metabolic Parameters.* Arteriosclerosis, Thrombosis, and Vascular Biology, 2007. **27**(10): p. 2250-2257.
18. Scherle, P., T. Behrens, and L.M. Staudt, *Ly-GDI, a GDP-dissociation inhibitor of the RhoA GTP-binding protein, is expressed preferentially in lymphocytes.* Proceedings of the National Academy of Sciences, 1993. **90**(16): p. 7568-7572.
19. Shi, M.K., Y.L. Xuan, and X.F. He, *FHL1 Overexpression as A Inhibitor of Lung Cancer Cell Invasion via Increasing RhoGDIß mRNA Expression.* Cell J, 2022. **24**(5): p. 239-244.
20. Kycia, I., B.N. Wolford, J.R. Huyghe, C. Fuchsberger, S. Vadlamudi, R. Kursawe, R.P. Welch, R.d.O. Albanus, A. Uyar, S. Khetan, et al., *A Common Type 2 Diabetes Risk Variant Potentiates Activity of an Evolutionarily Conserved Islet Stretch Enhancer and Increases C2CD4A and C2CD4B Expression.* The American Journal of Human Genetics, 2018. **102**(4): p. 620-635.
21. Ma, X., H. Zhao, J. Shan, F. Long, Y. Chen, Y. Chen, Y. Zhang, X. Han, and D. Ma, *PDCD10 interacts with Ste20-related kinase MST4 to promote cell growth and transformation via modulation of the ERK pathway.* Molecular biology of the cell, 2007. **18**(6): p. 1965-1978.
22. Maoz, A., M.A. Ciccone, S. Matsuzaki, R.L. Coleman, and K. Matsuo, *Emerging serine-threonine kinase inhibitors for treating ovarian cancer.* Expert Opinion on Emerging Drugs, 2019. **24**(4): p. 239-253.
23. Maoz, A., M.A. Ciccone, S. Matsuzaki, R.L. Coleman, and K. Matsuo, *Emerging serine-threonine kinase inhibitors for treating ovarian cancer.* Expert Opin Emerg Drugs, 2019. **24**(4): p. 239-253.
24. Saegusa, J., N. Akakura, C.-Y. Wu, C. Hoogland, Z. Ma, K.S. Lam, F.-T. Liu, Y.K. Takada, and Y. Takada, *Pro-inflammatory secretory phospholipase A2 type IIA binds to integrins alphavbeta3 and alpha4beta1 and induces proliferation of monocytic cells in an integrin-dependent manner.* Journal of Biological Chemistry, 2008. **283**(38): p. 26107-26115.
25. Wang, X., W. Mao, and X. Ma, *Integrin subunit alpha 5 maintains mitochondrial function in ox-LDL-induced cardiac microvascular endothelial cells via activating the PI3K/AKT signaling pathway.* Folia Morphologica, 2024.
26. Trusolino, L. and P.M. Comoglio, *Scatter-factor and semaphorin receptors: cell signalling for invasive growth.* Nature Reviews Cancer, 2002. **2**(4): p. 289-300.
27. Tao, Q., J. Fujimoto, T. Men, X. Ye, J. Deng, L. Lacroix, J.L. Clifford, L. Mao, C.S. Van Pelt, J.J. Lee, et al., *Identification of the Retinoic Acid–Inducible Gprc5a As a New Lung Tumor Suppressor Gene.* JNCI: Journal of the National Cancer Institute, 2007. **99**(22): p. 1668-1682.
28. Dai, J., Q. Li, Q. Zhou, S. Zhang, J. Chen, Y. Wang, J. Guo, Y. Gu, F. Gong, and Y. Tan, *IQCN disruption causes fertilization failure and male infertility due to manchette assembly defect.* EMBO Molecular Medicine, 2022. **14**(12): p. e16501.
29. Kleinboelting, S., A. Diaz, S. Moniot, J. van den Heuvel, M. Weyand, L.R. Levin, J. Buck, and C. Steegborn, *Crystal structures of human soluble adenylyl cyclase reveal mechanisms of catalysis and of its activation through bicarbonate.* Proceedings of the National Academy of Sciences, 2014. **111**(10): p. 3727-3732.
30. Schmid, A., Z. Sutto, M.-C. Nlend, G. Horvath, N. Schmid, J. Buck, L.R. Levin, G.E. Conner, N. Fregien, and M. Salathe, *Soluble adenylyl cyclase is localized to cilia and contributes to ciliary beat frequency regulation via production of cAMP.* The Journal of general physiology, 2007. **130**(1): p. 99-109.
31. Cabral-Dias, R., S. Lucarelli, K. Zak, S. Rahmani, G. Judge, J. Abousawan, L.F. DiGiovanni, D. Vural, K.E. Anderson, M.G. Sugiyama, et al., *Fyn and TOM1L1 are recruited to clathrin-coated pits and regulate Akt signaling.* Journal of Cell Biology, 2022. **221**(4): p. e201808181.
32. Keskitalo, S., E.M. Haapaniemi, V. Glumoff, X. Liu, V. Lehtinen, C. Fogarty, H. Rajala, S.C. Chiang, S. Mustjoki, P. Kovanen, et al., *Dominant TOM1 mutation associated with combined immunodeficiency and autoimmune disease.* npj Genomic Medicine, 2019. **4**(1): p. 14.
33. Yang, F., J. Ma, D. Zhu, Z. Wang, Y. Li, X. He, G. Zhang, and X. Kang, *The Role of S100A6 in Human Diseases: Molecular Mechanisms and Therapeutic Potential.* Biomolecules, 2023. **13**(7).
34. Ruhrberg, C., M.A. Hajibagheri, D.A. Parry, and F.M. Watt, *Periplakin, a novel component of cornified envelopes and desmosomes that belongs to the plakin family and forms complexes with envoplakin.* The Journal of cell biology, 1997. **139**(7): p. 1835-1849.
35. Hua, T., B.-b. Zhao, S.-b. Fan, C.-f. Zhao, Y.-h. Kong, R.-q. Tian, and B.-y. Zhang, *Prognostic implications of PPL expression in ovarian cancer.* Discover Oncology, 2022. **13**(1): p. 35.
36. Tian, Y., Y. Zhang, B. Zhong, Y.-Y. Wang, F.-C. Diao, R.-P. Wang, M. Zhang, D.-Y. Chen, Z.-H. Zhai, and H.-B. Shu, *RBCK1 Negatively Regulates Tumor Necrosis Factor- and Interleukin-1-triggered NF-κB Activation by Targeting TAB2/3 for Degradation **. Journal of Biological Chemistry, 2007. **282**(23): p. 16776-16782.
37. Pühringer, M., A. Eisenkölbl, and G. Gröppel, *Expanding the phenotype of RBCK1-associated polyglucosan body myopathy type 1.* Molecular Genetics and Metabolism Reports, 2024. **38**: p. 101031.

38. Block, J., C. Rashkova, I. Castanon, S. Zoghi, J. Platon, R.C. Ardy, M. Fujiwara, B. Chaves, R. Schoppmeyer, C.I. Van Der Made, et al., *Systemic Inflammation and Normocytic Anemia in DOCK11 Deficiency.* New England Journal of Medicine, 2023. **389**(6): p. 527-539.
39. Boussard, C., L. Delage, T. Gajardo, A. Kauskot, M. Batignes, N. Goudin, M.-C. Stolzenberg, C. Brunaud, P. Panikulam, Q. Riller, et al., *DOCK11 deficiency in patients with X-linked actinopathy and autoimmunity.* Blood, 2023. **141**(22): p. 2713-2726.
40. Alon, A., H.R. Schmidt, M.D. Wood, J.J. Sahn, S.F. Martin, and A.C. Kruse, *Identification of the gene that codes for the σ2 receptor.* Proceedings of the National Academy of Sciences, 2017. **114**(27): p. 7160-7165.
41. Masuda, T., T. Ishikawa, K. Mogushi, S. Okazaki, M. Ishiguro, S. Iida, H. Mizushima, H. Tanaka, H. Uetake, and K. Sugihara, *Overexpression of the S100A2 protein as a prognostic marker for patients with stage II and III colorectal cancer.* Int J Oncol, 2016. **48**(3): p. 975-82.
42. Studer, R.K., N. Vo, G. Sowa, C. Ondeck, and J. Kang, *Human nucleus pulposus cells react to IL-6: independent actions and amplification of response to IL-1 and TNF-α.* Spine (Phila Pa 1976), 2011. **36**(8): p. 593-9.
43. Gariano, G.R., V. Dell'Oste, M. Bronzini, D. Gatti, A. Luganini, M. De Andrea, G. Gribaudo, M. Gariglio, and S. Landolfo, *The intracellular DNA sensor IFI16 gene acts as restriction factor for human cytomegalovirus replication.* PLoS pathogens, 2012. **8**(1): p. e1002498.
44. Alimirah, F., J. Chen, H. Xin, and D. Choubey, *Androgen receptor auto-regulates its expression by a negative feedback loop through upregulation of IFI16 protein.* FEBS Lett, 2006. **580**(6): p. 1659-64.
45. Teske, N.C., J.Y. Engelen-Lee, S. Dyckhoff-Shen, H.W. Pfister, M. Klein, D. van de Beek, C.K. Kirschning, U. Koedel, and M.C. Brouwer, *The role of plasminogen activator inhibitor-2 in pneumococcal meningitis.* Acta Neuropathol Commun, 2022. **10**(1): p. 155.
46. Mezzadra, R., C. Sun, L.T. Jae, R. Gomez-Eerland, E. De Vries, W. Wu, M.E. Logtenberg, M. Slagter, E.A. Rozeman, and I. Hofland, *Identification of CMTM6 and CMTM4 as PD-L1 protein regulators.* Nature, 2017. **549**(7670): p. 106-110.
47. Burr, M.L., C.E. Sparbier, Y.-C. Chan, J.C. Williamson, K. Woods, P.A. Beavis, E.Y.N. Lam, M.A. Henderson, C.C. Bell, S. Stolzenburg, et al., *CMTM6 maintains the expression of PD-L1 and regulates anti-tumour immunity.* Nature, 2017. **549**(7670): p. 101-105.
48. Durinx, C., A.M. Lambeir, E. Bosmans, J.B. Falmagne, R. Berghmans, A. Haemers, S. Scharpé, and I. De Meester, *Molecular characterization of dipeptidyl peptidase activity in serum: soluble CD26/dipeptidyl peptidase IV is responsible for the release of X-Pro dipeptides.* European Journal of Biochemistry, 2000. **267**(17): p. 5608-5613.
49. Shin, J.W., G. Jurisic, and M. Detmar, *Lymphatic-specific expression of dipeptidyl peptidase IV and its dual role in lymphatic endothelial function.* Experimental Cell Research, 2008. **314**(16): p. 3048-3056.
50. Bishnoi, R., Y.-R. Hong, C. Shah, A. Ali, W.P. Skelton IV, J. Huo, N.H. Dang, and L.H. Dang, *Dipeptidyl peptidase 4 inhibitors as novel agents in improving survival in diabetic patients with colorectal cancer and lung cancer: A Surveillance Epidemiology and Endpoint Research Medicare study.* Cancer Medicine, 2019. **8**(8): p. 3918-3927.
51. Scholz, C.C., J. Rodriguez, C. Pickel, S. Burr, J.-a. Fabrizio, K.A. Nolan, P. Spielmann, M.A. Cavadas, B. Crifo, and D.N. Halligan, *FIH regulates cellular metabolism through hydroxylation of the deubiquitinase OTUB1.* PLoS biology, 2016. **14**(1): p. e1002347.
52. Hart, M., B. Walch-Rückheim, L. Krammes, T. Kehl, S. Rheinheimer, T. Tänzer, B. Glombitza, M. Sester, H.-P. Lenhof, and A. Keller, *miR-34a as hub of T cell regulation networks.* Journal for immunotherapy of cancer, 2019. **7**: p. 1-11.
53. Campbell, G.R., R.K. To, J. Hanna, and S.A. Spector, *SARS-CoV-2, SARS-CoV-1, and HIV-1 derived ssRNA sequences activate the NLRP3 inflammasome in human macrophages through a non-classical pathway.* iScience, 2021. **24**(4): p. 102295.
54. Elliott, Paul R., Sofie V. Nielsen, P. Marco-Casanova, Berthe K. Fiil, K. Keusekotten, N. Mailand, Stefan M.V. Freund, M. Gyrd-Hansen, and D. Komander, *Molecular Basis and Regulation of OTULIN-LUBAC Interaction.* Molecular Cell, 2014. **54**(3): p. 335-348.
55. Damgaard, R.B., J.A. Walker, P. Marco-Casanova, N.V. Morgan, H.L. Titheradge, P.R. Elliott, D. McHale, E.R. Maher, A.N.J. McKenzie, and D. Komander, *The Deubiquitinase OTULIN Is an Essential Negative Regulator of Inflammation and Autoimmunity.* Cell, 2016. **166**(5): p. 1215-1230.e20.
56. Tie, W. and F. Ge, *Lymphoid-specific helicase inhibits cervical cancer cells ferroptosis by promoting Nrf2 expression.* PeerJ, 2023. **11**: p. e16451.
57. Ozaki, K., T. Kuroki, S. Hayashi, and Y. Nakamura, *Isolation of Three Testis-Specific Genes (TSA303, TSA806, TSA903) by a Differential mRNA Display Method.* Genomics, 1996. **36**(2): p. 316-319.
58. Chen, H., H. You, L. Wang, X. Zhang, J. Zhang, and W. He, *Chaperonin-containing T-complex Protein 1 Subunit ζ Serves as an Autoantigen Recognized by Human Vδ2 γδ T Cells in Autoimmune Diseases.* Journal of Biological Chemistry, 2016. **291**(38): p. 19985-19993.
59. Granata, A., S.J. Koo, V. Haucke, G. Schiavo, and T.T. Warner, *CSN complex controls the stability of selected synaptic proteins via a torsinA-dependent process.* The EMBO Journal, 2011. **30**(1): p. 181-193-193.
60. Paquet, M.-E., M. Cohen-Doyle, G.C. Shore, and D.B. Williams, *Bap29/31 Influences the Intracellular Traffic of MHC Class I Molecules1.* The Journal of Immunology, 2004. **172**(12): p. 7548-7555.
61. Li, H., Q. Wang, and P. Lyu, *Chimeric RNAs and their implication in prostate cancer.* Cancer Pathogenesis and Therapy, 2023. **01**(03): p. 216-219.
62. Liu, J., Z. Chen, P. Zhao, and W. Li, *Prognostic and immune regulating roles of YIF1B in Pan-Cancer: a potential target for both survival and therapy response evaluation.* Bioscience Reports, 2020. **40**(7).

63. Tsunedomi, R., K. Yoshimura, Y. Kimura, M. Nishiyama, N. Fujiwara, S. Matsukuma, S. Kanekiyo, H. Matsui, Y. Shindo, Y. Watanabe, et al., *Elevated expression of RAB3B plays important roles in chemoresistance and metastatic potential of hepatoma cells.* BMC Cancer, 2022. **22**(1): p. 260.
64. Mintoo, M., V. Rajagopalan, and J.P. O'Bryan, *Intersectin — many facets of a scaffold protein.* Biochemical Society Transactions, 2024. **52**(1): p. 1-13.
65. Vollweiter, D., J.K. Shergill, A. Hilse, G. Kochlamazashvili, S.P. Koch, S. Mueller, P. Boehm-Sturm, V. Haucke, and T. Maritzen, *Intersectin deficiency impairs cortico-striatal neurotransmission and causes obsessive–compulsive behaviors in mice.* Proceedings of the National Academy of Sciences, 2023. **120**(35): p. e2304323120.
66. Zhai, L.-L., P.-P. Qiao, Y.-S. Sun, T.-F. Ju, and Z.-G. Tang, *Tumorigenic and immunological roles of Heat shock protein A2 in pancreatic cancer: a bioinformatics analysis.* Revista da Associação Médica Brasileira, 2022. **68**(4): p. 470-475.
67. Say, Y.-H., Y.Y. Sio, A.H.S. Heng, Y.T. Ng, S.A. Matta, S.L. Pang, K.F. Teh, Y.R. Wong, S.M. Rawanan Shah, K. Reginald, et al., *Golgin A7 family member B (GOLGA7B) is a plausible novel gene associating high glycaemic index diet with acne vulgaris.* Experimental Dermatology, 2022. **31**(8): p. 1208-1219.
68. Zhou, L., S. Dong, Y. Deng, P. Yang, Y. Zheng, L. Yao, M. Zhang, S. Yang, Y. Wu, Z. Zhai, et al., *GOLGA7 rs11337, a Polymorphism at the MicroRNA Binding Site, Is Associated with Glioma Prognosis.* Molecular Therapy Nucleic Acids, 2019. **18**: p. 56-65.
69. Kravtsova-Ivantsiv, Y., I. Shomer, V. Cohen-Kaplan, B. Snijder, G. Superti-Furga, H. Gonen, T. Sommer, T. Ziv, A. Admon, I. Naroditsky, et al., *KPC1-Mediated Ubiquitination and Proteasomal Processing of NF-κB1 p105 to p50 Restricts Tumor Growth.* Cell, 2015. **161**(2): p. 333-347.
70. Kamura, T., T. Hara, M. Matsumoto, N. Ishida, F. Okumura, S. Hatakeyama, M. Yoshida, K. Nakayama, and K.I. Nakayama, *Cytoplasmic ubiquitin ligase KPC regulates proteolysis of p27(Kip1) at G1 phase.* Nat Cell Biol, 2004. **6**(12): p. 1229-35.
71. Bhardwaj, A. and K. Van Steen, *Multi-omics Data and Analytics Integration in Ovarian Cancer*. 2020, Springer International Publishing. p. 347-357.
72. Zhang, R., X. Hou, C. Wang, J. Li, J. Zhu, Y. Jiang, and F. Hou, *The Endoplasmic Reticulum ATP13A1 is Essential for MAVS-Mediated Antiviral Innate Immunity.* Adv Sci (Weinh), 2022. **9**(33): p. e2203831.
73. He, Y., S. Alejo, J.D. Johnson, S. Jayamohan, and G.R. Sareddy, *Reticulocalbin 3 Is a Novel Mediator of Glioblastoma Progression.* Cancers, 2023. **15**(7): p. 2008.
74. He, Y., S. Alejo, J.D. Johnson, S. Jayamohan, and G.R. Sareddy, *Reticulocalbin 3 Is a Novel Mediator of Glioblastoma Progression.* Cancers (Basel), 2023. **15**(7).
75. Hall, R., V. Sawant, J. Gu, T. Sikora, B. Rollo, S. Velasco, J. Kim, N. Segev, J. Christodoulou, and N.J. Van Bergen, *TRAPPopathies: Severe Multisystem Disorders Caused by Variants in Genes of the Transport Protein Particle (TRAPP) Complexes.* International Journal of Molecular Sciences, 2024. **25**(24): p. 13329.
76. Fan, Y., Z. Si, L. Wang, and L. Zhang, *DYT-TOR1A dystonia: an update on pathogenesis and treatment.* Frontiers in Neuroscience, 2023. **17**.
77. Haas, S., A. Steplewski, L.D. Siracusa, S. Amini, and K. Khalili, *Identification of a Sequence-specific Single-stranded DNA Binding Protein That Suppresses Transcription of the Mouse Myelin Basic Protein Gene (*).* Journal of Biological Chemistry, 1995. **270**(21): p. 12503-12510.
78. Zhang, P., J.-H. Zhao, L. Chen, Z.-L. Bian, L.-L. Ju, H.-X. Wang, and W.-H. Cai, *Expression and function of myelin expression factor 2 in hepatocellular carcinoma.* BMC Gastroenterology, 2023. **23**(1): p. 20.
79. Lin, C.-C., Y.-R. Shen, C.-C. Chang, X.-Y. Guo, Y.-Y. Young, T.-Y. Lai, I.S. Yu, C.-Y. Lee, T.-H. Chuang, H.-Y. Tsai, et al., *Terminal uridyltransferase 7 regulates TLR4-triggered inflammation by controlling Regnase-1 mRNA uridylation and degradation.* Nature Communications, 2021. **12**(1): p. 3878.
80. Brandt, D.T., C. Baarlink, T.M. Kitzing, E. Kremmer, J. Ivaska, P. Nollau, and R. Grosse, *SCAI acts as a suppressor of cancer cell invasion through the transcriptional control of β1-integrin.* Nature cell biology, 2009. **11**(5): p. 557-568.
81. Gasparics, Á., G. Kökény, A. Fintha, R. Bencs, M.M. Mózes, E.I. Ágoston, A. Buday, Z. Ivics, P. Hamar, B. Győrffy, et al., *Alterations in SCAI Expression during Cell Plasticity, Fibrosis and Cancer.* Pathology & Oncology Research, 2018. **24**(3): p. 641-651.
82. Treiber, T., N. Treiber, U. Plessmann, S. Harlander, J.L. Daiß, N. Eichner, G. Lehmann, K. Schall, H. Urlaub, and G. Meister, *A Compendium of RNA-Binding Proteins that Regulate MicroRNA Biogenesis.* Mol Cell, 2017. **66**(2): p. 270-284.e13.
83. Tiurin, V.I., E.V. Preobrazhenskaya, N.V. Mitiushkina, A.A. Romanko, A.A. Anuskina, R.S. Mulkidjan, E.S. Saitova, E.A. Krivosheyeva, E.D. Kharitonova, M.P. Shevyakov, et al., *Rapid and Cost-Efficient Detection of RET Rearrangements in a Large Consecutive Series of Lung Carcinomas.* Int J Mol Sci, 2023. **24**(13).
84. Popow, J., J. Jurkin, A. Schleiffer, and J. Martinez, *Analysis of orthologous groups reveals archease and DDX1 as tRNA splicing factors.* Nature, 2014. **511**(7507): p. 104-107.
85. Zhang, R., M.V. Poustovoitov, X. Ye, H.A. Santos, W. Chen, S.M. Daganzo, J.P. Erzberger, I.G. Serebriiskii, A.A. Canutescu, R.L. Dunbrack, et al., *Formation of MacroH2A-Containing Senescence-Associated Heterochromatin Foci and Senescence Driven by ASF1a and HIRA.* Developmental Cell, 2005. **8**(1): p. 19-30.
86. Hsu, C.J., O. Meers, M. Buschbeck, and F.H. Heidel, *The Role of MacroH2A Histone Variants in Cancer.* Cancers, 2021. **13**(12).
87. Li, R., H. Pei, D.K. Watson, and T.S. Papas, *EAP1/Daxx interacts with ETS1 and represses transcriptional activation of ETS1 target genes.* Oncogene, 2000. **19**(6): p. 745-753.

88. Yordy, J.S., R. Li, V.I. Sementchenko, H. Pei, R.C. Muise-Helmericks, and D.K. Watson, *SP100 expression modulates ETS1 transcriptional activity and inhibits cell invasion.* Oncogene, 2004. **23**(39): p. 6654-6665.
89. Adlat, S., R.K. Sah, F. Hayel, Y. Chen, F.B. Bah, M. Al-Azab, N. Bahadar, M. Myint, Z.M. Oo, M.I. Nasser, et al., *Global transcriptome study of Dip2B-deficient mouse embryonic lung fibroblast reveals its important roles in cell proliferation and development.* Computational and Structural Biotechnology Journal, 2020. **18**: p. 2381-2390.
90. Hergeth, S.P. and R. Schneider, *The H1 linker histones: multifunctional proteins beyond the nucleosomal core particle.* EMBO reports, 2015. **16**(11): p. 1439-1453.
91. Warneboldt, J., F. Haller, O. Horstmann, B.C. Danner, L. Füzesi, D. Doenecke, and N. Happel, *Histone H1x is highly expressed in human neuroendocrine cells and tumours.* BMC Cancer, 2008. **8**(1): p. 388.
92. Nabeel-Shah, S., S. Pu, J.D. Burns, U. Braunschweig, N. Ahmed, G.L. Burke, H. Lee, E. Radovani, G. Zhong, H. Tang, et al., *C2H2-zinc-finger transcription factors bind RNA and function in diverse post-transcriptional regulatory processes.* Molecular Cell, 2024. **84**(19): p. 3810-3825.e10.
93. Safra, M., R. Nir, D. Farouq, I. Vainberg Slutskin, and S. Schwartz, *TRUB1 is the predominant pseudouridine synthase acting on mammalian mRNA via a predictable and conserved code.* Genome research, 2017. **27**(3): p. 393-406.
94. Kurimoto, R., T. Chiba, Y. Ito, T. Matsushima, Y. Yano, K. Miyata, Y. Yashiro, T. Suzuki, K. Tomita, and H. Asahara, *The tRNA pseudouridine synthase TruB1 regulates the maturation of let-7 miRNA.* The EMBO Journal, 2020. **39**(20): p. e104708.
95. Chu, J., J. Jiang, X. Fan, J. Liu, K. Gao, Y. Jiang, M. Li, W. Xi, L. Zhang, K. Bian, et al., *A novel MYC-ZNF706-SLC7A11 regulatory circuit contributes to cancer progression and redox balance in human hepatocellular carcinoma.* Cell Death & Differentiation, 2024. **31**(10): p. 1333-1348.
96. Gudimchuk, N.B. and J.R. McIntosh, *Regulation of microtubule dynamics, mechanics and function through the growing tip.* Nature Reviews Molecular Cell Biology, 2021. **22**(12): p. 777-795.
97. Di Donato, N., A.E. Timms, K.A. Aldinger, G.M. Mirzaa, J.T. Bennett, S. Collins, C. Olds, D. Mei, S. Chiari, G. Carvill, et al., *Analysis of 17 genes detects mutations in 81% of 811 patients with lissencephaly.* Genetics in Medicine, 2018. **20**(11): p. 1354-1364.
98. Mundel, P., H.W. Heid, T.M. Mundel, M. Krüger, J. Reiser, and W. Kriz, *Synaptopodin: An Actin-associated Protein in Telencephalic Dendrites and Renal Podocytes.* Journal of Cell Biology, 1997. **139**(1): p. 193-204.
99. Huang, L., B. Wei, Y. Zhao, X. Gong, and L. Chen, *DYNLT1 promotes mitochondrial metabolism to fuel breast cancer development by inhibiting ubiquitination degradation of VDAC1.* Molecular Medicine, 2023. **29**(1): p. 72.
100. Tang, H., Y. Wang, B. Zhang, S. Xiong, L. Liu, W. Chen, G. Tan, and H. Li, *High brain acid soluble protein 1(BASP1) is a poor prognostic factor for cervical cancer and promotes tumor growth.* Cancer Cell International, 2017. **17**(1): p. 97.
101. Verdonschot, J.A.J., E.K. Vanhoutte, G.R.F. Claes, A.T.J.M. Helderman-van den Enden, J.G.J. Hoeijmakers, D.M.E.I. Hellebrekers, A. de Haan, I. Christiaans, R.H. Lekanne Deprez, H.M. Boen, et al., *A mutation update for the FLNC gene in myopathies and cardiomyopathies.* Human Mutation, 2020. **41**(6): p. 1091-1111.
102. Zhang, H., Y.-z. Pan, M. Cheung, M. Cao, C. Yu, L. Chen, L. Zhan, Z.-w. He, and C.-y. Sun, *LAMB3 mediates apoptotic, proliferative, invasive, and metastatic behaviors in pancreatic cancer by regulating the PI3K/Akt signaling pathway.* Cell Death & Disease, 2019. **10**(3): p. 230.
103. Tong, Y., H. Yamaguchi, E. Giaime, S. Boyle, R. Kopan, R.J. Kelleher, and J. Shen, *Loss of leucine-rich repeat kinase 2 causes impairment of protein degradation pathways, accumulation of α-synuclein, and apoptotic cell death in aged mice.* Proceedings of the National Academy of Sciences, 2010. **107**(21): p. 9879-9884.
104. Rust, M.B., S. Khudayberdiev, S. Pelucchi, and E. Marcello, *CAPt'n of Actin Dynamics: Recent Advances in the Molecular, Developmental and Physiological Functions of Cyclase-Associated Protein (CAP).* Frontiers in Cell and Developmental Biology, 2020. **8**: p. 586631.
105. Xu, X., F. Duan, L. Xu, S. Ng, Y. Li, Y. Li, X. Wang, T. Long, N. Ding, and E. Xu, *High expression of AFAP1-AS1 is associated with poor prognosis of digestive system cancers: A meta-analysis.* Medicine, 2022. **101**(38): p. e30833.
106. Chen, P.-W. and G.S. Kroog, *Leupaxin is similar to paxillin in focal adhesion targeting and tyrosine phosphorylation but has distinct roles in cell adhesion and spreading.* Cell Adhesion & Migration, 2010. **4**(4): p. 527-540.
107. Bonaud, A., S. Clare, V. Bisio, J.M. Sowerby, S. Yao, H. Ostergaard, K. Balabanian, K.G.C. Smith, and M. Espéli, *Leupaxin Expression Is Dispensable for B Cell Immune Responses.* Frontiers in Immunology, 2020. **11**: p. 466.
108. Larrucea, S., N. Butta, E.G. Arias-Salgado, S. Alonso-Martin, M.S. Ayuso, and R. Parrilla, *Expression of podocalyxin enhances the adherence, migration, and intercellular communication of cells.* Experimental Cell Research, 2008. **314**(10): p. 2004-2015.
109. Zhang, H., R. Zhang, J. Yao, X. Hu, Y. Pu, S. He, J. Yu, H. Zhu, B. Mu, and C. Zhao, *Effect of F11R Gene Knockdown on Malignant Biological Behaviors of Pancreatic Cancer Cells.* Journal of Oncology, 2022. **2022**(1): p. 3379027.
110. Katz, M., I. Amit, A. Citri, T. Shay, S. Carvalho, S. Lavi, F. Milanezi, L. Lyass, N. Amariglio, J. Jacob-Hirsch, et al., *A reciprocal tensin-3-cten switch mediates EGF-driven mammary cell migration.* Nature Cell Biology, 2007. **9**(8): p. 961-U124.
111. Mainsiouw, L., M.E. Ryan, S. Hafizi, and J.C. Fleming, *The molecular and clinical role of Tensin 1/2/3 in cancer.* J Cell Mol Med, 2023. **27**(13): p. 1763-1774.
112. Thapa, C., P. Roivas, T. Haataja, P. Permi, and U. Pentikäinen, *Interaction mechanism of endogenous PP2A inhibitor protein ENSA with PP2A.* The FEBS Journal, 2022. **289**(2): p. 519-534.
113. Daniel, H.-A., K. Youngjo, S. Min Sup, and S. and Su Jung, *New Insights into the Role of E2s in the Pathogenesis of Diseases: Lessons Learned from UBE2O.* Molecules and Cells, 2018. **41**(3): p. 168-178.

114. Pan, Y.-R., J.C.-Y. Lai, W.-K. Huang, P.-H. Peng, S.-M. Jung, S.-H. Lin, C.-P. Chen, C.-E. Wu, T.-H. Hung, A.L. Yu, et al., *PLK1 and its substrate MISP facilitate intrahepatic cholangiocarcinoma progression by promoting lymphatic invasion and impairing E-cadherin adherens junctions.* Cancer Gene Therapy, 2024. **31**(2): p. 322-333.
115. Plešingerová, H., P. Janovská, A. Mishra, L. Smyčková, L. Poppová, A. Libra, K. Plevová, P. Ovesná, L. Radová, M. Doubek, et al., *Expression of COBLL1 encoding novel ROR1 binding partner is robust predictor of survival in chronic lymphocytic leukemia.* Haematologica, 2018. **103**(2): p. 313-324.
116. Li, Y., Y. Chen, B. Shao, J. Liu, R. Hu, F. Zhao, X. Cui, X. Zhao, and Y. Wang, *Evaluation of creatine kinase (CK)-MB to total CK ratio as a diagnostic biomarker for primary tumors and metastasis screening.* Practical Laboratory Medicine, 2023. **37**: p. e00336.
117. Najt, C.P., S. Senthivinayagam, M.B. Aljazi, K.A. Fader, S.D. Olenic, J.R.L. Brock, T.A. Lydic, A.D. Jones, and B.P. Atshaves, *Liver-specific loss of Perilipin 2 alleviates diet-induced hepatic steatosis, inflammation, and fibrosis.* American Journal of Physiology-Gastrointestinal and Liver Physiology, 2016. **310**(9): p. G726-G738.
118. Pourteymour, S., S. Lee, T.M. Langleite, K. Eckardt, M. Hjorth, C. Bindesbøll, K.T. Dalen, K.I. Birkeland, C.A. Drevon, T. Holen, et al., *Perilipin 4 in human skeletal muscle: localization and effect of physical activity.* Physiol Rep, 2015. **3**(8): p. e12481.
119. Chen, T.J., B.H. Hsu, S.W. Lee, C.C. Yang, Y.F. Tian, Y.H. Kuo, W.S. Li, H.H. Tsai, L.C. Wu, C.F. Yeh, et al., *Overexpression of Dehydrogenase/Reductase 9 Predicts Poor Response to Concurrent Chemoradiotherapy and Poor Prognosis in Rectal Cancer Patients.* Pathol Oncol Res, 2022. **28**: p. 1610537.
120. Hamid, A.R.A.H., M.J. Pfeiffer, G.W. Verhaegh, E. Schaafsma, A. Brandt, F.C.G.J. Sweep, J.P.M. Sedelaar, and J.A. Schalken, *Aldo-keto Reductase Family 1 Member C3 (AKR1C3) Is a Biomarker and Therapeutic Target for Castration-Resistant Prostate Cancer.* Molecular Medicine, 2012. **18**(11): p. 1449-1455.
121. Matsumoto, R., M. Tsuda, K. Yoshida, M. Tanino, T. Kimura, H. Nishihara, T. Abe, N. Shinohara, K. Nonomura, and S. Tanaka, *Aldo-keto reductase 1C1 induced by interleukin-1β mediates the invasive potential and drug resistance of metastatic bladder cancer cells.* Scientific Reports, 2016. **6**(1): p. 34625.
122. Shi, M., X.-R. Nan, and B.-Q. Liu, *The Multifaceted Role of FUT8 in Tumorigenesis: From Pathways to Potential Clinical Applications.* International Journal of Molecular Sciences, 2024. **25**(2): p. 1068.
123. Burgh, R.v.d., J. Meeldijk, L. Jongeneel, J. Frenkel, N. Bovenschen, M.v. Gijn, and M. Boes, *Reduced serpinB9-mediated caspase-1 inhibition can contribute to autoinflammatory disease.* Oncotarget, 2016. **7**(15).
124. Smith, M.A., V. Mack, A. Ebneth, I. Moraes, B. Felicetti, M. Wood, D. Schonfeld, O. Mather, A. Cesura, and J. Barker, *The structure of mammalian serine racemase: evidence for conformational changes upon inhibitor binding.* Journal of Biological Chemistry, 2010. **285**(17): p. 12873-12881.
125. Ishizuka, T., I. Fujimori, M. Kato, C. Noji-Sakikawa, M. Saito, Y. Yoshigae, K. Kubota, A. Kurihara, T. Izumi, T. Ikeda, et al., *Human Carboxymethylenebutenolidase as a Bioactivating Hydrolase of Olmesartan Medoxomil in Liver and Intestine.* Journal of Biological Chemistry, 2010. **285**(16): p. 11892-11902.
126. Huang, Y., C. Xiong, C. Wang, J. Deng, Z. Zuo, H. Wu, J. Xiong, X. Wu, H. Lu, Q. Hao, et al., *p53-responsive CMBL reprograms glucose metabolism and suppresses cancer development by destabilizing phosphofructokinase PFKP.* Cell Reports, 2023. **42**(11): p. 113426.
127. Khan, N., P.P. Shah, D. Ban, P. Trigo-Mouriño, M.G. Carneiro, L. DeLeeuw, W.L. Dean, J.O. Trent, L.J. Beverly, M. Konrad, et al., *Solution structure and functional investigation of human guanylate kinase reveals allosteric networking and a crucial role for the enzyme in cancer.* Journal of Biological Chemistry, 2019. **294**(31): p. 11920-11933.
128. Hidalgo-Gutierrez, A., J. Shintaku, J. Ramon, E. Barriocanal-Casado, A. Pesini, R.P. Saneto, G. Garrabou, J.C. Milisenda, A. Matas-Garcia, L. Gort, et al., *Guanylate Kinase 1 Deficiency: A Novel and Potentially Treatable Mitochondrial DNA Depletion/Deletions Disease.* Annals of Neurology, 2024. **96**(6): p. 1209-1224.
129. Basu, S.S., M. Basu, Z. Li, and S. Basu, *Characterization of Two Glycolipid:α2-3Sialyltransferases, SAT-3 (CMP-NeuAc:nLcOse4Cer α2-3sialyltransferase) and SAT-4 (CMP-NeuAc:GgOse4Cer α2-3sialyltransferase), from Human Colon Carcinoma (Colo 205) Cell Line.* Biochemistry, 1996. **35**(16): p. 5166-5174.
130. Chen, X., W. Su, J. Chen, P. Ouyang, and J. Gong, *ST3GAL4 promotes tumorigenesis in breast cancer by enhancing aerobic glycolysis.* Human Cell, 2024. **38**(1): p. 1.
131. Wang, Y., S. Fan, J. Lu, Z. Zhang, D. Wu, Z. Wu, and Y. Zheng, *GLUL Promotes Cell Proliferation in Breast Cancer.* J Cell Biochem, 2017. **118**(8): p. 2018-2025.
132. Xu, Z., Y. Sun, J. Jiang, and P. Liu, *The role of KIAA1191 in the necroptotic pathway of multiple myeloma.* Annals of Hematology, 2022. **101**(2): p. 359-367.
133. Zhu, H., S. Blake, K.T. Chan, R.B. Pearson, and J. Kang, *Cystathionine β-Synthase in Physiology and Cancer.* BioMed Research International, 2018. **2018**(1): p. 3205125.
134. Bombarda-Rocha, V., D. Silva, A. Badr-Eddine, P. Nogueira, J. Gonçalves, and P. Fresco, *Challenges in Pharmacological Intervention in Perilipins (PLINs) to Modulate Lipid Droplet Dynamics in Obesity and Cancer.* Cancers, 2023. **15**(15): p. 4013.

biomolecules

Original blots

Fig. 1B

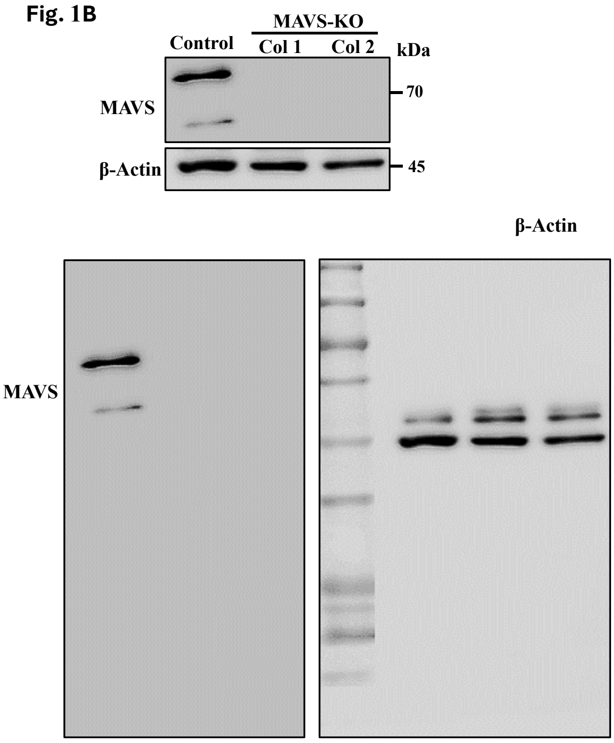

Fig. 2C

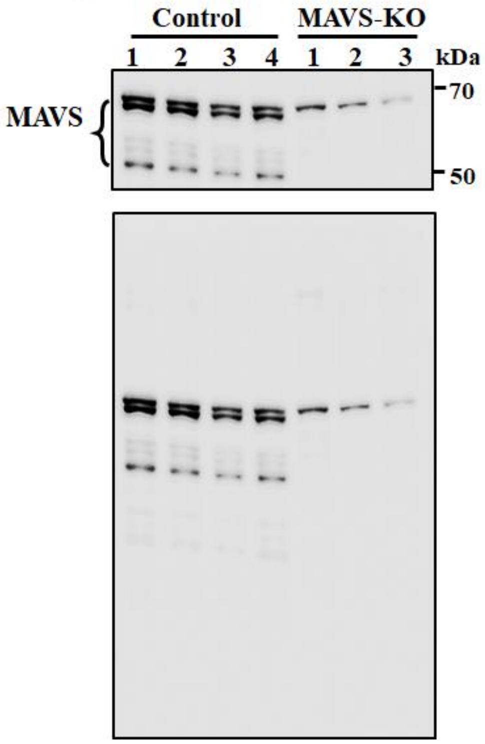

Fig. 4C

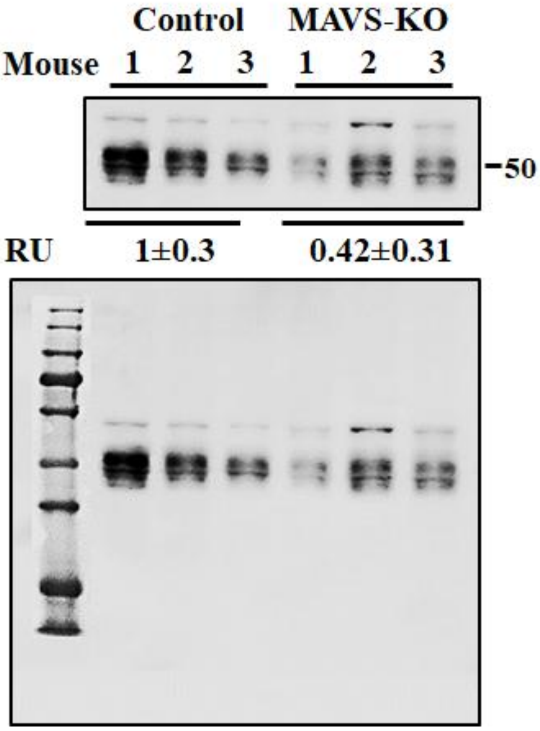

Fig. 6G

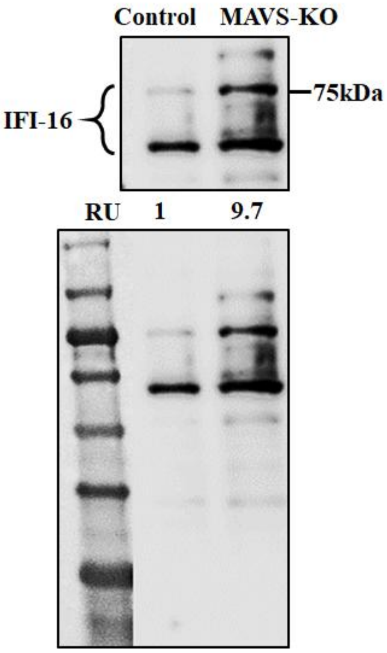

Supplement: Supplementary file 1 [file biomolecules-16-00501-s001.zip › biomolecules-4188009-supplementary.pdf]
